# Secondary Metabolism in the Gill Microbiota of Shipworms (Teredinidae) as Revealed by Comparison of Metagenomes and Nearly Complete Symbiont Genomes

**DOI:** 10.1128/mSystems.00261-20

**Published:** 2020-06-30

**Authors:** Marvin A. Altamia, Zhenjian Lin, Amaro E. Trindade-Silva, Iris Diana Uy, J. Reuben Shipway, Diego Veras Wilke, Gisela P. Concepcion, Daniel L. Distel, Eric W. Schmidt, Margo G. Haygood

**Affiliations:** a Ocean Genome Legacy Center, Department of Marine and Environmental Science, Northeastern University, Nahant, Massachusetts, USA; b The Marine Science Institute, University of the Philippines Diliman, Quezon City, Philippines; c Department of Medicinal Chemistry, University of Utah, Salt Lake City, Utah, USA; d Bioinformatic and Microbial Ecology Laboratory—BIOME, Federal University of Bahia, Salvador, Bahia, Brazil; e Drug Research and Development Center, Department of Physiology and Pharmacology, Federal University of Ceará, Ceará, Brazil; f Philippine Genome Center, University of the Philippines Diliman, Quezon City, Philippines; g Institute of Marine Science, School of Biological Sciences, University of Portsmouth, Portsmouth, United Kingdom; University of Vienna

**Keywords:** biosynthesis, metagenomics

## Abstract

Shipworms play critical roles in recycling wood in the sea. Symbiotic bacteria supply enzymes that the organisms need for nutrition and wood degradation. Some of these bacteria have been grown in pure culture and have the capacity to make many secondary metabolites. However, little is known about whether such secondary metabolite pathways are represented in the symbiont communities within their hosts. In addition, little has been reported about the patterns of host-symbiont co-occurrence. Here, we collected shipworms from the United States, the Philippines, and Brazil and cultivated symbiotic bacteria from their gills. We analyzed sequences from 22 shipworm gill metagenomes from seven shipworm species and from 23 cultivated symbiont isolates. Using (meta)genome sequencing, we demonstrate that the cultivated isolates represent all the major bacterial symbiont species and strains in shipworm gills. We show that the bacterial symbionts are distributed among shipworm hosts in consistent, predictable patterns. The symbiotic bacteria harbor many gene cluster families (GCFs) for biosynthesis of bioactive secondary metabolites, only <5% of which match previously described biosynthetic pathways. Because we were able to cultivate the symbionts and to sequence their genomes, we can definitively enumerate the biosynthetic pathways in these symbiont communities, showing that ∼150 of ∼200 total biosynthetic gene clusters (BGCs) present in the animal gill metagenomes are represented in our culture collection. Shipworm symbionts occur in suites that differ predictably across a wide taxonomic and geographic range of host species and collectively constitute an immense resource for the discovery of new biosynthetic pathways corresponding to bioactive secondary metabolites.

**IMPORTANCE** We define a system in which the major symbionts that are important to host biology and to the production of secondary metabolites can be cultivated. We show that symbiotic bacteria that are critical to host nutrition and lifestyle also have an immense capacity to produce a multitude of diverse and likely novel bioactive secondary metabolites that could lead to the discovery of drugs and that these pathways are found within shipworm gills. We propose that, by shaping associated microbial communities within the host, the compounds support the ability of shipworms to degrade wood in marine environments. Because these symbionts can be cultivated and genetically manipulated, they provide a powerful model for understanding how secondary metabolism impacts microbial symbiosis.

## Abstract (precis)

We define a system in which the major symbionts that are important to host biology and to the production of secondary metabolites can be cultivated. We show that symbiotic bacteria that are critical to host nutrition and lifestyle also have an immense capacity to produce a multitude of diverse and likely novel bioactive secondary metabolites that could lead to the discovery of drugs and that these pathways are found within shipworm gills. We propose that, by shaping associated microbial communities within the host, the compounds support the ability of shipworms to degrade wood in marine environments. Because these symbionts can be cultivated and genetically manipulated, they provide a powerful model for understanding how secondary metabolism impacts microbial symbiosis.

## INTRODUCTION

**FIG 1:**
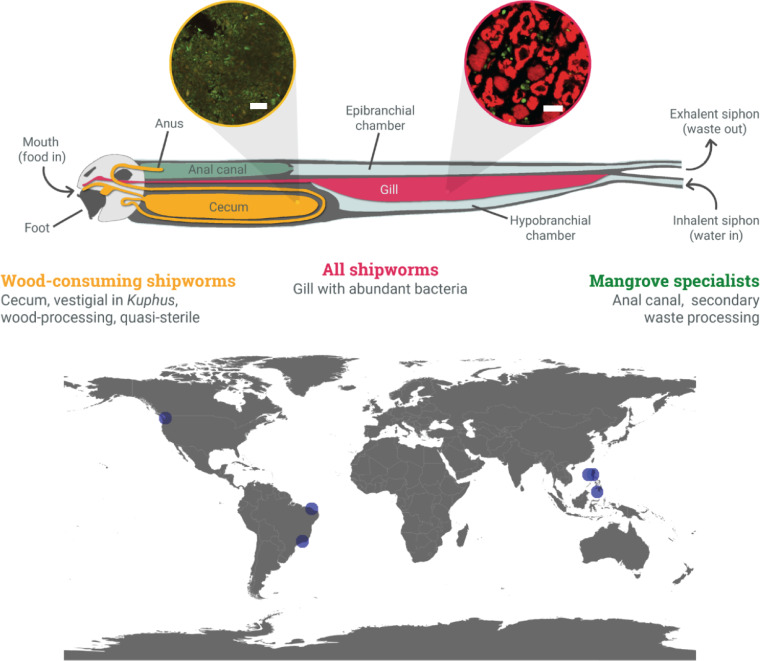
(Top) Diagram of generic shipworm anatomy. Insets are from Fig. 2, panels B and D, in Betcher et al. (8). Bars, 20 μm. Red, signal from a fluorescent universal bacterial probe, indicating large numbers of bacterial symbionts in the bacteriocytes of the gill and paucity of bacteria in the cecum; green, background fluorescence. (Bottom) Collection locations of specimens included in this study. See Table S1 for details.

Shipworms (family Teredinidae) are bivalve mollusks found throughout the world’s oceans (1, 2). Many shipworms eat wood, assisted by cellulases from the intracellular symbiotic gammaproteobacteria that inhabit their gills (Fig. 1) (3–6). Other shipworms use sulfide metabolism, also relying on gill-dwelling gammaproteobacteria for sulfur oxidation (7). Shipworm gill symbionts of several different species are thus essential to shipworm nutrition and survival. One of the most remarkable features of the shipworm system is that wood digestion does not take place where the bacteria are located, such that the bacterial cellulase products are transferred from the gill to a nearly sterile cecum (8), where wood digestion occurs (Fig. 1) (9). This enables the host shipworms to directly consume glucose and other sugars derived from wood lignocellulose rather than from the less-energetic-fermentation by-products of cellulolytic gut microbes as found in other symbioses. Shipworm symbionts are also essential for the nitrogen fixation that helps to offset the low nitrogen content of wood (10, 11). Thus, shipworms have evolved structures and mechanisms enabling bacterial metabolism that supports animal host nutrition.

10.1128/mSystems.00261-20.7 TABLE S1 (A) Shipworm gill metagenomes used in this study. (B) Shipworm symbiont genomes. (C) Statistics of genomic bins from metagenomes generated by checkM. Download Table S1, DOCX file, 0.04 MB. Copyright © 2020 Altamia et al. 2020 Altamia et al. This content is distributed under the terms of the Creative Commons Attribution 4.0 International license.

While the bacteria in many nutritional symbioses are difficult to cultivate, shipworm gill symbiotic gammaproteobacteria have been brought into stable culture (5, 12, 13). This led to the discovery that these bacteria are exceptional sources of secondary metabolites (14). Of bacteria with sequenced genomes, the gill symbiont Teredinibacter turnerae T7901 and related strains are among the richest sources of biosynthetic gene clusters (BGCs), comparable in content to well-known producers of commercial importance such as *Streptomyces* spp. (13–16). This implies that shipworms might be a good source of new compounds for drug discovery. Of equal importance, the symbiotic bacteria are crucial to survival of host shipworms, and bioactive secondary metabolites might play a role in shaping those symbioses.

An early analysis of the T. turnerae T7901 genome revealed nine complex polyketide synthase (PKS) and nonribosomal peptide synthetase (NRPS) BGCs (14). One of these was shown to produce a novel catecholate siderophore, turnerbactin, which is crucial in obtaining iron and for the survival of the symbiont in which it may be crucial for obtaining iron both within the host and in the external environment (17). A second BGC synthesizes borated polyketide tartrolons D and E, which are antibiotic and potently antiparasitic compounds (18). Both were detected in the extracts of shipworms, implying a potential role in producing the remarkable near-sterility observed in the cecum (8). These data suggested specific roles for secondary metabolism in shipworm ecology.

T. turnerae T7901 is just one of multiple strains and species of gammaproteobacteria living intracellularly in shipworm gills (3, 12), and thus these analyses just begin to describe shipworm secondary metabolism. Many shipworm species are generalists, consuming wood from a variety of sources (1, 19). Other wood-eating shipworms, such as Dicyathifer mannii, Bactronophorus thoracites, and Neoteredo reynei, are specialists that live in the submerged branches, trunks, and rhizomes of mangroves (20, 21). There, they play an important role in ecological processes in mangrove ecosystems, i.e., transferring large amounts of carbon fixed by mangroves to the marine environment (19). Several shipworm species, such as Kuphus polythalamius, live in other substrates. For example, K. polythalamius is often found in sediment habitats (as well as in wood) where its gill symbionts are crucial to sulfide oxidation and carry out carbon fixation (7, 22). K. polythalamius lacks significant amounts of cellulolytic symbionts such as T. turnerae and instead contains Thiosocius teredinicola, which oxidizes sulfide and generates energy for the host (23). Other shipworms are found in solid rock and in seagrass (24, 25). Thus, gill symbionts vary, but in all cases the symbionts appear to be essential to the survival of shipworms.

While the potential of T. turnerae as an unexplored producer of secondary metabolites has been described previously (14, 16), the capacity of other shipworm symbionts is still largely unknown. Moreover, several findings indicate that the BGCs found in cultivated isolates might also be harbored by symbionts within shipworm gills. Previous obtained data include the detection of tartrolons and turnerbactins and their BGCs in shipworms (17, 18) and results of an investigation of four isolate genomes and one metagenome (26); also, an exploratory investigation of the metagenome of N. reynei gills and digestive tract led to the detection of known T. turnerae BGCs as well as novel clusters (27). These findings left unaddressed many major issues concerning shipworm secondary metabolites, including which symbionts make them, how they are distributed, how they vary by host and symbiont species, and what their roles might be in nature.

To address those issues, we used comparative metagenomics, selecting six species of wood-eating shipworms (B. thoracites, N. reynei, Bankia setacea, *Bankia* sp., D. mannii, and *Teredo* sp.), comparing these to a seventh sulfide-oxidizing group, *Kuphus* spp. We compared gill metagenomes from 22 specimens comprising seven animal species with the genomes of 23 cultivated bacteria isolated from shipworms. These isolated bacteria included 22 cellulolytic and sulfur-oxidizing isolates cultivated from shipworm tissue samples. By comparing the gill metagenomes to isolate strain genomes, we demonstrate that the cultivated bacterial genomes accurately represent the genomes of symbionts found in the gills, and we show that they share many of the same secondary metabolic BGCs. Moreover, we show that the members of symbiont communities differ among shipworm species, indicating that surveying more host shipworms will lead to discovery of new BGCs and new bacterial symbionts.

## RESULTS AND DISCUSSION

### Sequencing data.

Most of the genomes and metagenomes are described here for the first time, or, in a few cases, previously reported genomes/metagenomes were resequenced/reassembled/reanalyzed (see Materials and Methods). Two bacterial genomes of T. turnerae T7901 and T. teredinicola 2141T and metagenomes of K. polythalamius were previously described (7, 14, 23). The resulting statistics and accession numbers are provided in Table S1A in the supplemental material, while specimen and strain origins, many of which had not been previously reported, are given in Table S1B. For the bacterial strains, six of the circular genomes were closed, while remaining assemblies had between 2 and 141 scaffolds. Metagenome total assembly sizes ranged from 2.6 × 10^8^ to 1.3 × 10^9^ bp, with *N*_50_ values of 860 to 4,530 bp. The highest *N*_50_ values were obtained with the Philippines specimens sequenced at the University of Utah, while others sequenced elsewhere had comparatively lower *N*_50_ values.

### Mapping gill metagenomes to cultivated bacteria.

**FIG 2:**
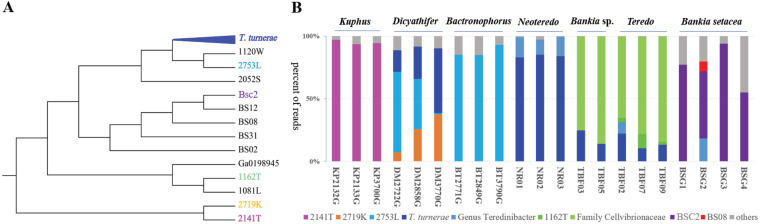
Cultivated bacterial isolates represent the major shipworm gill symbionts. (A) Isolated bacteria analyzed in this study are shown in abstracted schematic of a 16S rRNA phylogenetic tree. The complete tree with accurate branch lengths and bootstrap numbers is shown in Fig. S1. T. turnerae comprised 11 sequenced strains; for other groups, individual strains are shown. Each color indicates different bacteria appearing in the metagenomes in panel B. (B) Species composition of shipworm gill symbiont community based on shotgun metagenome sequence analysis. The *y*-axis data indicate the percentages of reads mapping to each bacterial species, while the *x*-axis data indicate the individual shipworm specimens used in the study. Colors indicate the origin of bacterial reads; gray represents minor, sporadic, unidentified strains.

A phylogenetic tree created from the 16S rRNA genes of the cultivated bacteria (Fig. 2A; see also Fig. S1 in the supplemental material) revealed that the strains were all gammaproteobacteria. Of these, the two sulfur oxidizers were members of the order *Chromatiales* (*Thiosocius* and allies) whereas the remaining 21 were strains of cellulolytic bacteria that were members of the order *Cellvibrionales*, including 11 strains of T. turnerae and 10 strains of diverse cellulolytic bacteria. With the exception of T. turnerae and strain Bs02, which was recently formally described as a new species, Teredinibacter waterburyi (28), most of these bacterial species had not been previously described.

10.1128/mSystems.00261-20.1 FIG S1 Phylogeny of shipworm gill symbionts and related free-living bacteria based on approximate maximum-likelihood tree of 16S rRNA sequences. The tree was reconstructed using 1,125 nucleotide positions employing the GTR substitution model in FastTree version 2.1.11 with optimized Gamma20 likelihood and rate categories per site set to a value of 20. Support values are indicated for each node. The scale bar represents the nucleotide substitution rate per site. Cultivated shipworm symbionts and related bacteria are indicated in boldface. An excerpted version of this tree is shown in Fig. 2A. Download FIG S1, EPS file, 0.3 MB. Copyright © 2020 Altamia et al. 2020 Altamia et al. This content is distributed under the terms of the Creative Commons Attribution 4.0 International license.

**FIG 3:**
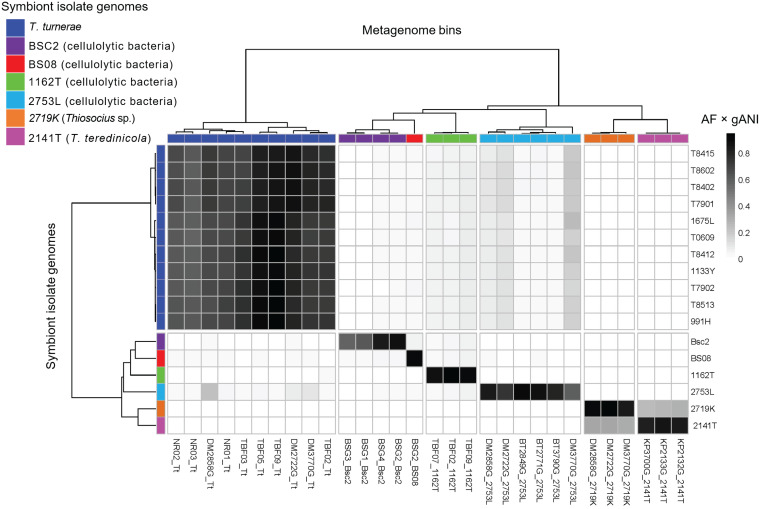
Heat map of relationships between symbiont isolate genomes and gill metagenome bins. The scale bar is shaded according to identity on the basis of calculated values (AF × gANI). Color bars in the phylogenetic tree indicate bacterial species identity, either in the metagenomes or in the genome, and they are identical to the codes shown in Fig. 2. This figure indicates the high degree of certainty that the cultivated isolates are the same species as the major bacteria present in the gill.

Further, whole-genome-based average nucleotide identity (gANI) measurements reinforce the 16S rRNA-based phylogenetic tree of sequenced strains (Fig. 2 and 3; see also Fig. S1 and Table S2 in the supplemental material). Previously proposed cutoffs for bacterial species differentiation suggest that bacterial strains with gANI values of ≥0.95 are conspecific, although several well-known species have lower gANI values (29). The concatenated T. turnerae strains are represented by two groups, exemplified by strains T7901 and T7902 (Fig. S2). In comparisons within each group, the T. turnerae strains were found to have gANI values of >0.97, whereas the gANI values determined in between-group comparisons were ∼0.92. This agrees with and reinforces a previously published observation that T. turnerae is comprised of two distinct clades associated with different host species and suggests that these clades may in fact constitute distinct but closely related bacterial species (12). Outside T. turnerae, the strains are much less closely related, with alignment fraction (AF) × genome average nucleotide identity (gANI) values of <0.4 (Fig. 3; see also Fig. S2), indicating that they are all different at the species level.

10.1128/mSystems.00261-20.2 FIG S2 AF × gANI comparison reveals species-level differences. The heat map shows AF × gANI values comparing strain isolate genomes to each other. This analysis shows that T. turnerae forms 2 distinct groups, which may possibly represent different species. However, the other isolates are much more distantly related, with AF × gANI scores usually <0.2. Sulfide-oxidizing bacteria also bear some similarity. Download FIG S2, EPS file, 0.7 MB. Copyright © 2020 Altamia et al. 2020 Altamia et al. This content is distributed under the terms of the Creative Commons Attribution 4.0 International license.

10.1128/mSystems.00261-20.8 TABLE S2 gANI comparison of genomes and metagenome bins. Download Table S2, DOCX file, 0.2 MB. Copyright © 2020 Altamia et al. 2020 Altamia et al. This content is distributed under the terms of the Creative Commons Attribution 4.0 International license.

The bacteria living in gills were grouped into bins that represent individual species of bacteria (Fig. 2B). For example, in *Kuphus* spp., >95% of bacterial reads could be mapped to cultivated isolate strain T. teredinicola 2141T. Among the three specimens measured, 14 bins mapped to T. teredinicola 2141T (Table S2). None of the other specimens in our study had any match to T. teredinicola 2141T, with gANI values of >0.90. Normalized by length, these bins had a total gANI value = 0.96 (Table 1). In combination with the results of phylogenetic analyses, these data suggest that T. teredinicola 2141T is conspecific with the uncultivated symbionts in the metagenomes of *Kuphus* spp.

**TABLE 1:** Example gANI values for shipworm gills in comparison to sequenced isolates, extracted from Table S2 data

Comparison	Total metagenomic bin size (bp)	gANI
*Kuphus* spp. to *T. teredicincola* 2141T	23,758,169	0.963839
*D. mannii* and *B. thoracites* to 2753L	26,758,239	0.990273
*D. mannii* to 2719K	14,895,102	0.992012
*B. setacea* to BSC2	19,687,153	0.974108
*Teredo* sp. to 1162T	1,489,154	0.984036
*B. setacea* to BS08	3,513,639	0.995137

Similarly, *Cellvibrionaceae* strain 2753L was mapped to 20 bins in D. mannii and B. thoracites specimens, with a total gANI value of >0.99. Mapping bins to discrete strains as shown in Fig. 2B, the gANI was 0.96 to 0.99 to a single strain, with much lower identity to other strains sequenced. These data demonstrate a high level of identity between cultivated isolates and the strains present within shipworm gills, suggesting that in some cases these strains were nearly identical to those present within the shipworms.

In other cases, either because we had multiple strains representing a species (as in the case of T. turnerae) or because the identity to single strains was not as pronounced, we described bins as “T. turnerae,” “genus *Teredinibacter*,” and “family *Cellvibrionaceae*.” These still had relatively high identities to cultivated isolates. For example, the *Teredo* sp. bins in total had a gANI of >0.98 to cultivated isolates in our strain collection. It is likely that the metagenomes from these animals were not as similar to those of the cultivated isolates because, in those cases, we compared metagenomes of isolates from Philippines specimens with metagenomes of isolates from Brazilian animals.

In sum, these data demonstrate conclusively that the cultivated isolates obtained from shipworm gills accurately represent the strains found within shipworms. The data suggest that the isolates are the same species as the naturally occurring symbionts in the animals and that in many cases their DNA sequences are >99% identical at the whole-genome level. More than 85% of the DNA in each specimen’s gill metagenome is represented by a cultivated isolate in our collection (with the exception of one specimen), and the remaining <15% of the DNA belongs to multiple, low-abundance species, most of which are not reproducibly found among isolates from different shipworm specimens. Further, the shipworm literature focuses on the readily cultivable species T. turnerae. We show that T. turnerae is dominant in some shipworm species but that it is present at very low levels or even absent in others.

### Strain variation increases genetic diversity of shipworm microbiota.

Metagenome binning showed that each gill contained 1 to 3 major bacterial species. Since we had data representing deep sequencing of the major metagenomic bacterial species, we expected to provide complete assemblies. Previously, using similarly deep data, we had obtained relatively complete assemblies or even assembled whole bacterial genomes from metagenomes (30). Here, however, our metagenome bin *N*_50_ values were only in the very low thousands.

Investigating why the assembly was difficult, we noted that we often obtained very similar contigs with different copy numbers. For example, a single metagenome bin containing Bsc2-like contigs is shown in Table S3. The pairwise identities between contigs were between 93% and 98% in DNA sequence, indicating that these bins were comprised of mixtures of very closely related bacteria. We saw a very similar phenomenon in a recent investigation of K. polythalamius symbionts (7). In that case, the strains were nearly identical and could not be resolved by analysis of 16S rRNA gene sequences, which were 100% identical. Thus, we developed a different method to quantify the strain-level variation that was observed using metagenomics.

10.1128/mSystems.00261-20.9 TABLE S3 Comparison of contigs in the Bsc2 bin. Download Table S3, DOCX file, 0.2 MB. Copyright © 2020 Altamia et al. 2020 Altamia et al. This content is distributed under the terms of the Creative Commons Attribution 4.0 International license.

In the *Kuphus* study, we cut the DNA gyrase B gene into 50-bp segments and aligned single reads to each 50-bp segment (7). By quantifying the reads for each observed single nucleotide polymorphism (SNP), we confirmed that the gill symbiont species consisted of several strains, and we quantified their relative abundances. Here, we investigated the major strains found in the remaining shipworm species using the same method and show four representative examples in Fig. S3. This analysis showed that similar levels of strain variation represent a widespread phenomenon in shipworm gills and that such variation is not just restricted to K. polythalamius. Strain variation is an important source of BGC variation, as described below.

10.1128/mSystems.00261-20.3 FIG S3 Strain variation among GCFs in shipworm gill symbiont bacterial species. This analysis was performed as previously reported for *Kuphus* symbionts (7), using DNA gyrase B in 50-bp frames and examining SNP variation. Different colors indicate reads with different SNPs along the gyrase sequence. The *y*-axis data represent numbers of reads observed, while the *x*-axis data indicate each 50-bp region. Download FIG S3, EPS file, 4.9 MB. Copyright © 2020 Altamia et al. 2020 Altamia et al. This content is distributed under the terms of the Creative Commons Attribution 4.0 International license.

### Discovery and analysis of BGCs.

**FIG 4:**
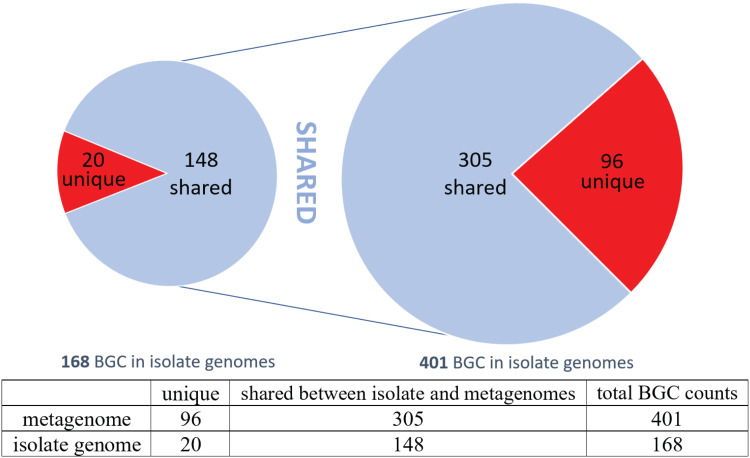
Most BGCs found in the metagenomes and in the bacterial isolate genomes are shared. A total of 401 BGCs from metagenome sequences were compared to the bacterial isolate genomes, 305 of which were found in isolates. Conversely, 148 of 168 BGCs from sequenced bacterial isolates were found in the metagenomes. The shared numbers likely differ because the contigs assembled from the metagenome sequences were shorter on average such that several metagenome fragments can map to a single BGC in an isolate.

We compared secondary metabolic pathways in the isolates and in the animal specimens. An inventory of the BGC content performed using antiSMASH (31) revealed a large number of BGCs: 431 BGCs were identified in the 23 cultivated isolates alone. Because raw antiSMASH output includes many hypothetical or poorly characterized BGCs, we focused on well-characterized classes of secondary metabolic proteins and pathways: polyketide synthases (PKSs), nonribosomal peptide synthetases (NRPSs), siderophores, terpenes, homoserine lactones, and thiopeptides. Using these criteria, we identified 168 BGCs from 23 cultivated isolates and 401 BGCs from 22 shipworm gill metagenomes (Fig. 4). Because the genomes of cultivated isolates were well assembled, we could discern and analyze entire BGCs. In contrast, gill metagenomes had smaller contigs such that the BGCs were fragmented.

The BGCs identified in this study originated nearly universally from the cellulolytic *Cellvibrionales* strains, with very few BGCs found in the sulfide-oxidizing strains of *Chromatiales*. We found only five BGCs that were similar to previously identified clusters outside shipworms, based upon >70% of genes conserved in antiSMASH. The remainder appeared to be unknown or uncharacterized BGCs. In turn, the new BGCs are likely to represent new compounds, while the characterized BGCs represent those corresponding to previously identified compounds. In addition, it is possible that some of the new BGCs may represent known compounds for which biosynthetic pathways have not yet been discovered.

**FIG 5:**
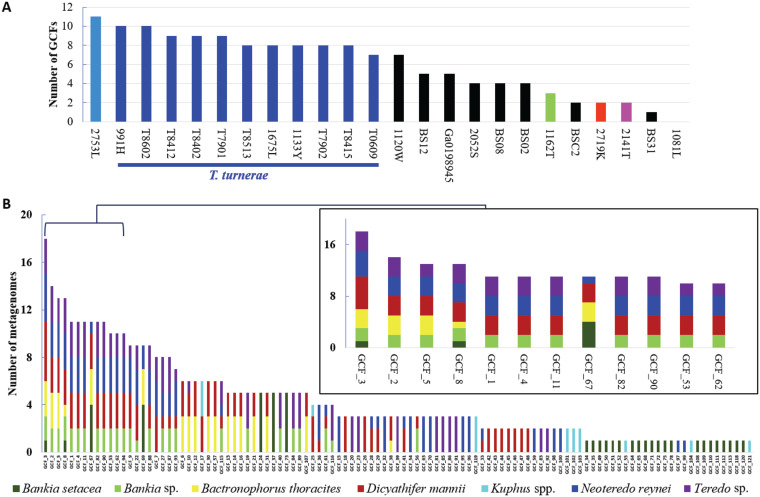
GCFs found in (A) bacterial genomes and (B) gill metagenomes. (A) A list of strains of cultivated bacterial genomes is provided in the *x* axis, while the number of total GCFs in different sequenced strains is shown in the *y* axis. Colors indicate bacteria as described for Fig. 2A. Because there were 11 isolates of T. turnerae, the levels of GCFs in this group (dark blue bars) are comparatively overrepresented in the diagram. (B) GCFs (*x* axis) found in each metagenome (*y* axis) are shown. The inset expands a region containing the most common GCFs found in our specimens. Colors indicate shipworm host species. See Table S4 for a complete list of GCFs used in this figure.

To facilitate comparison between metagenomes, we grouped all 569 BGCs into 122 gene cluster families (GCFs), where each GCF was comprised of closely related BGCs (32, 33) (Fig. 5; see also Table S4). BGCs grouped into a single GCF are highly likely to encode the production of identical or closely related secondary metabolites.

10.1128/mSystems.00261-20.10 TABLE S4 List of GCFs found in this study. Download Table S4, DOCX file, 0.2 MB. Copyright © 2020 Altamia et al. 2020 Altamia et al. This content is distributed under the terms of the Creative Commons Attribution 4.0 International license.

**FIG 6:**
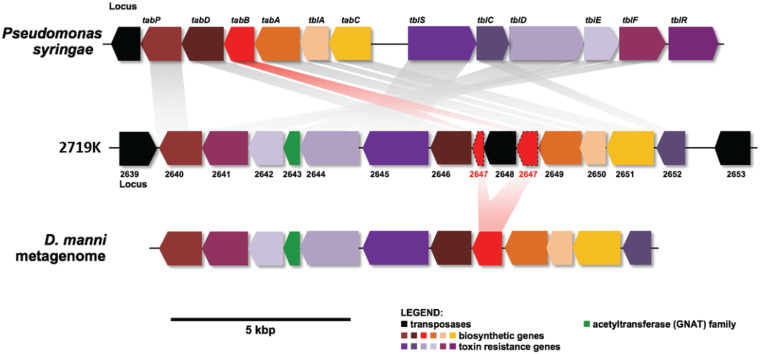
A possible tabtoxin pathway is found in the D. mannii metagenome. Tabtoxin is a phytotoxin β-lactam that was initially discovered in *Pseudomonas* spp. (top). Strain 2719K contained a tabtoxin-like cluster that was pseudogenized (shown as an insertion in *tabB*; middle). A nonpseudogenized tabtoxin-like cluster was found in the D. mannii metagenome gill (bottom), supporting the observation that multiple variants of each symbiont genome are represented in each metagenome.

**FIG 7:**
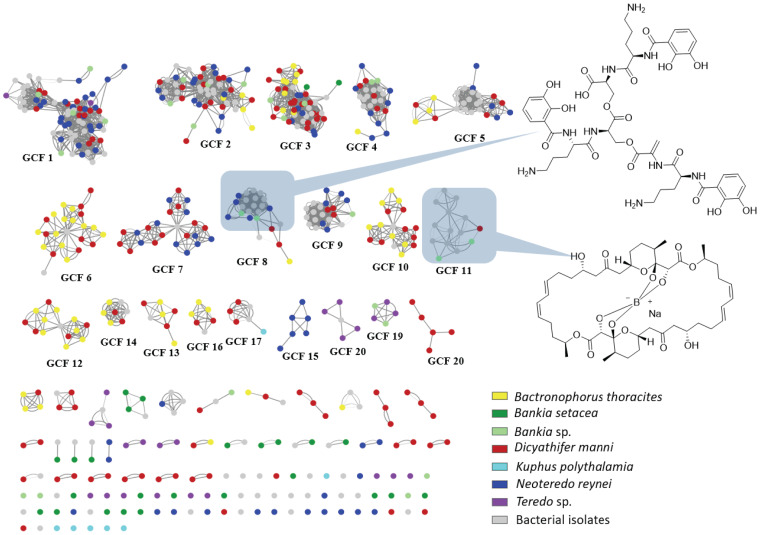
GCF distribution across shipworm species. Shown is a similarity network diagram, in which circles indicate individual BGCs from sequenced isolates (gray) and gill metagenomes (colors indicate species of origin; see legend). Lines indicate the MultiGeneBlast scores from comparisons between identified BGCs, with thinner lines indicating a lower degree of similarity. For example, the cluster labeled “GCF_8” encodes the pathway for the siderophore turnerbactin, the structure of which is shown at the right. The main cluster, circled by a light blue box, includes BGCs that are very similar to the originally described turnerbactin gene cluster. More distantly related BGCs, with fewer lines connecting them to the majority nodes in GCF_8, might represent other siderophores. The GCF_11 data likely all represent tartrolon D/E, a boronated polyketide shown at the right. For detailed alignments of BGCs, see Fig. S4.

Some important BGCs were excluded using our method. One of these is worth describing briefly, since it illustrates a major limitation of the methods that we describe and is potentially important for symbiosis. In the genome of *Chromatiales* strain 2719K, we discovered a gene cluster for tabtoxin (34, 35) or a related compound (Fig. 6). This cluster does not contain common PKS/NRPS elements and thus is not one of the GCFs shown in Fig. 5, 7, or 8. A key biosynthetic gene in the tabtoxin-like cluster was pseudogenous in strain 2719K, but the D. mannii gill metagenome contained an apparently functional pathway. Tabtoxin is an important β-lactam that is used by *Pseudomonas* in plant pathogenesis (36, 37).

**FIG 8:**
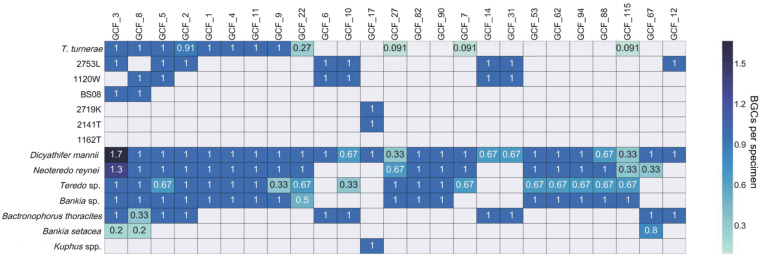
Integration of tBLASTn and networking analyses reveals the pattern of occurrence of GCFs in isolates and metagenomes. Here, we show only the most commonly occurring GCFs. The values in each box indicate the number of BGC occurrences per specimen for each GCF (see Fig. S5 for details). When the number equals 1, then the BGC is found in all specimens of that species. When the number is less than 1, it then indicates the fraction of specimens in which the pathway is found. A number greater than 1 is specific to GCF_3, for which two different types are possible (see Fig. 8). In that case, there were two different classes of GCF_3 in two D. mannii specimens and one N. reynei specimen and only one class in the other specimens.

10.1128/mSystems.00261-20.4 FIG S4 Representative alignments showing the actual data underlying the clusters shown in Fig. 4, 5, 7, and 8. (A) Representative alignment of GCF_3 from genomes and metagenomes. Three subtypes are indicated by red, blue, and green coloring; for example, the NR03 metagenome contains two copies of the blue subtype. DM2858G and DM2722G contain blue and red subtypes. (B) Alignment of GCF_2. (C) Alignment of GCF_5. (D) Alignment of GCF_8. Download FIG S4, EPS file, 6.8 MB. Copyright © 2020 Altamia et al. 2020 Altamia et al. This content is distributed under the terms of the Creative Commons Attribution 4.0 International license.

10.1128/mSystems.00261-20.5 FIG S5 Occurrence of GCFs in individual samples, expanding what is shown in Fig. 8. (A) GCFs found in bacterial strains. (B) GCFs from individual shipworm specimens. Download FIG S5, EPS file, 6.1 MB. Copyright © 2020 Altamia et al. 2020 Altamia et al. This content is distributed under the terms of the Creative Commons Attribution 4.0 International license.

### Comparison of isolate and gill BGCs.

Of 401 BGCs identified in the metagenomes, 305 had close relatives in cultivated isolates, indicating that ∼75% of BGCs in the metagenomes are covered in our sequenced culture collection (Fig. 4). Conversely, among 168 isolate BGCs, 148 (90%) were found in the metagenomes. Thus, sequencing of additional cultivated isolates in our strain collections would likely yield additional novel BGCs. Since the 11 T. turnerae strains analyzed in this project contained different BGCs, we speculate that the additional BGC variation is due to the observed strain variation in the shipworm gills.

It is difficult to quantify BGCs in metagenomes, which usually contain relatively small contigs. Since the BGC classes analyzed were >10 kbp in length, each BGC was usually represented by multiple, short contigs, which are not easily linked. Here, we had an advantage in that the cultivated isolates accurately represented the gill metagenomes; thus, we were able to map the identified metagenomic contigs to the assembled BGCs found in cultivated isolates.

Using this mapping, we accurately estimated the number of unique BGCs in the gill symbiont community. For example, 305 metagenome BGCs were found to be synonymous with 148 isolate BGCs, indicating that the metagenome BGC count can be estimated to be approximately double the actual number of BGCs. To verify this estimate, we selected GCFs 2, 3, 5, and 8, aligning the metagenomic contigs against the BGCs from cultivated isolates (Fig. S4). In the metagenomes, of the total of 401 BGCs identified, 100 were members of these four GCFs, but some of them were just fragments of the full-length BGCs found in cultivated isolates. When the 100 metagenomic BGCs were aligned to their congeners in cultivated isolates, they could be collapsed into 46 unique BGCs. Thus, using two different approaches, we were able to estimate that the 401 metagenomic BGCs of all GCFs represented ∼200 actual BGCs in the shipworm gills. To the best of our knowledge, this has not been possible for other metagenomes/symbioses and represents a powerful aspect of this system.

Most GCFs detected were unique or nearly so, occurring in only one or two of the examined strains (Fig. 7 and 8). Only 8 GCFs were found to be distributed in 10 or more isolates, and these mostly represented pathways that are universal or nearly universal in T. turnerae, which was overrepresented in our data set. In contrast, in the metagenomes, most of the 107 GCFs were found in multiple specimens. Forty-five GCFs were found in multiple species of shipworms. Sixty-two GCFs were found in only a single shipworm species; 26 of these were found only in a single specimen (Fig. 5). These data demonstrate that accessing diverse shipworm specimens, as well as diverse shipworm species, will lead to the discovery of many novel BGCs. In addition, this result reinforces the data representing the strain-level variation found in shipworms revealed both in the metagenome assembly results and in the DNA gyrase B SNP analysis.

We used MultiGeneBlast (32) output to construct a GCF similarity network (Fig. 7), which provides an easily interpretable diagram of how GCFs are distributed among bacteria. However, a notable shortcoming was observed. In a long-term drug discovery campaign, we have found the tartrolon BGC in nearly all T. turnerae strains (18) (unpublished observation). However, this BGC was observed via MultiGeneBlast in only a few of the T. turnerae-hosting shipworms. This result was a consequence of the presence of the repetitive sequences found in large *trans*-acyltransferase (*trans*-AT) pathways that can hinder assembly (38). Thus, we were concerned that networking might underreport the similarity of some types of biosynthetic pathways.

To remedy this problem, we obtained GCFs from cultivated isolates and searched them against metagenome contigs using tBLASTn (Fig. 8). This provided an orthogonal view of secondary metabolism in shipworms, revealing the presence of the tartrolon pathway, as well as of other pathways that do not assemble well in metagenomes because of characteristics such as repetitive DNA sequences. A weakness of this second method is that it does not tell us whether two pathways are related closely enough to encode the production of similar compounds. Thus, these two methods provide different insights into BGCs in shipworm gills.

The high level of similarity of BGCs between cultivated isolates and metagenomes further reinforced the species identities determined by gANI (Table 1). Since secondary metabolism is often one of the most variable genomic features in bacteria, the sharing of multiple pathways between gills and isolates provides further evidence that the isolates are representative of the true symbionts found in gills.

We identified three categories of GCFs: (i) GCFs that are widely shared among shipworm species, (ii) GCFs that were specific to select shipworm and symbiont species, and (iii) GCFs that were distributed among specimens without an obvious relationship to host or symbiont species identity. These pathways are described in the following sections.

**(i) Widely shared GCFs.** Four pathways (GCF_2, GCF_3, GFC_5, and GCF_8) were prevalent in all wood-eating shipworms, regardless of sample location (Fig. 7 and 8). These GCFs were harbored in the genomes of T. turnerae, the most widely distributed shipworm symbiont, and those of several other *Cellvibrionales* symbiont isolates from wood eating shipworms (especially pathway-rich isolate 2753L).

**FIG 9:**
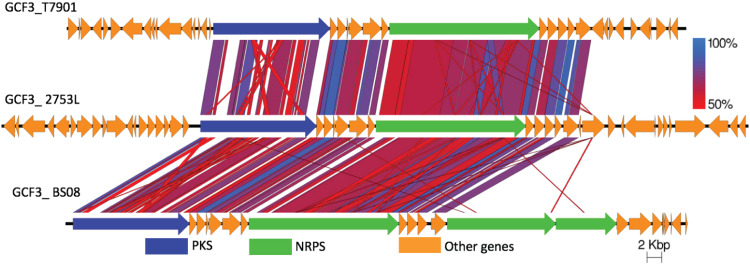
Three types of GCF_3 gene clusters were found to be distributed in all cellulolytic shipworms in this study. tBLASTx was used to compare the clusters, demonstrating the presence of three closely related GCF_3 gene families in all cellulolytic shipworm gills.

The most widely occurring pathway in shipworm gill metagenomes is GCF_3. It was identified in all gill metagenomes with cellulolytic symbionts, including the metagenome of specimen B, setacea Bsc2. It occurred in all T. turnerae strains, as well as in *Cellvibrionales* strains 2753L and Bs08. It was first annotated as “region 1” in the T. turnerae T7901 genome and encodes an elaborate hybrid *trans*-AT PKS-NRPS pathway (14). Unlike all other GCFs identified in shipworm metagenomes and isolates, GCF_3 could be subdivided into at least three discrete categories, all of which included different gene content (Fig. 9). The first category, identified in T. turnerae T7901, encodes a PKS and a single NRPS, in addition to several potential modifying enzymes. In strain Bs08, instead of just a single NRPS, GCF_3 contains three NRPS genes. Presumably, Bs08 and T7901 produce products with similar or identical polyketides and amino acids, except that Bs08 adds two more amino acids to the chain. *Cellvibrionales* 2753L encoded the third pathway type, which was similar to that found in T7901 except with different flanking genes that might encode modifying enzymes. Thus, T7901 and 2753L might make identical or very similar polyketide-peptide scaffolds which are modified slightly differently after scaffold synthesis. The presence of a single GCF that encodes similar but nonidentical products suggests dynamic pathway evolution within shipworms.

GCF_2 encodes a NRPS/*trans*-AT PKS pathway, the chemical products of which are unknown. It is found in all shipworm specimens in this study and in all T. turnerae strains. It is also present in *Cellvibrionales* strain 2753L. This explains its presence in B. thoracites despite the absence of T. turnerae in this species. GCF_2 is synonymous with “region 3” described in the annotation of the T. turnerae T7901 genome (14).

GCF_5 harbors a combination of terpene cyclase and predicted arylpolyene biosynthetic genes (39). Although the cyclase and surrounding regions have all of the genes necessary to make and export hopanoids, the GCF_5 biosynthetic product is unknown. In addition to occurring in all T. turnerae strains, GCF_5 is present in *Cellvibrionales* strains 1120W and 2753L. The pathway was detected in all wood-eating specimens except *Teredo* sp. TBF07 (Fig. 9).

GCF_8 is exemplified by the previously described turnerbactin BGC, from T. turnerae T7901. Turnerbactin is a catecholate siderophore, crucial to iron acquisition in T. turnerae (17). The BGC for turnerbactin was identified and described as “region 7” in the previously published T. turnerae T7901 genome. GCF_8 was found to be present in all T. turnerae genomes sequenced here. Other *Cellvibrionales* strains, including 2753L from B. thoracites and Bs08 from B. setacea (neither of which contains T. turnerae), also encode turnerbactin-like siderophore synthesis. GCF_8 was also found in the metagenome of one specimen of B. thoracites. Beyond bacterial iron acquisition, siderophores are also important in strain competition and potentially in host animal physiology (40, 41), possibly explaining the widespread distribution of GCF_8. From the clustering pattern in Fig. 7, it seems likely that GCF_8 comprises at least three different but related types of gene clusters. Thus, GCF_8 likely represents catecholate siderophores but not necessarily turnerbactin.

**(ii) Bacterial species-specific GCFs.** CFs 1, 4, and 11 were found in all T. turnerae-containing shipworms. GCF_1 is a *trans*-AT PKS-NRPS pathway that appears to be split into two clusters in some shipworm isolates, including T. turnerae T7901, in which it was previously annotated as “region 4” and “region 5.” GCF_4 is the previously described “region 8” PKS-NRPS from T. turnerae T7901. Most notably, GCF_11 encodes tartrolon biosynthesis (18). Tartrolon is an antibiotic and potent antiparasitic agent isolated from culture broths of T. turnerae T7901 (18, 42, 43). It has also been identified in the cecum of the shipworm. It was proposed previously that the bacteria synthesize tartrolon in the gill and that it is transferred to the cecum, where it may play a role in keeping the digestive tract free of bacteria (18).

The gill metagenomes of D. mannii and B. thoracites indicate the abundant presence of 2753L-like strains. Like T. turnerae T7901, the 2753L isolate genome includes GCFs 2, 3, and 5. However, 2753L contains several GCFs not found in T. turnerae, including GCFs 6, 10, 12, 13, 14, 16, 30, and 31 (listed in order of their relative frequencies of occurrence in samples). These GCFs are also evident in D. mannii and B. thoracites gill metagenomes. These are PKS and NRPS clusters that lack close relatives according to antiSMASH and thus may synthesize novel secondary metabolite classes.

Brazilian shipworms *Bankia* sp. and *Teredo* sp. contain T. turnerae, but they are dominated by symbiont genomes from other symbiotic *Cellvibrionaceae* bacteria. Although those species are not represented in our current culture collection, they are closely related to isolate 1162T from a Philippine specimen of *Lyrodus* sp. The metagenomes of *Bankia* sp. and *Teredo* sp. contain many GCFs that were not found in the sequenced isolates (Fig. S5B). In addition, the GCFs found in *Bankia* sp. and *Teredo* sp. do not overlap completely, implying that the *Cellvibrionaceae* bacteria found in these different host species are distinct. A high number of GCFs were found, indicating that the symbionts might potentially have GCF content similar to that of GCF-rich isolate 2753L.

The B. setacea specimens shown in Fig. S5B were found to contain pathways specifically found in *Cellvibrionaceae* isolate Bsc2, which was the major bacterium observed in the B. setacea gill metagenome sequences.

The K. polythalamius gill metagenome and its cultivated sulfur-oxidizing symbiont T. teredinicola were found to contain relatively few BGCs, but strikingly, two NRPS-containing GCFs were found in all shipworm specimens containing the sulfide-oxidizing symbionts (K. polythalamius and D. mannii) and all sulfide-oxidizing symbiont isolates (T. teredinicola and isolate 2719K). One of these, GCF_17, is shown in Fig. 8. Thus, it is clear that the BGCs contained by the cellulolytic symbionts are more abundant and diverse.

**(iii) GCFs for which patterns of occurrence are not obviously related to host species identity.** Overall, the most abundant pathways in shipworms were identical to those seen in the corresponding cultivated bacterial symbionts (Fig. 7 and 8). Since the pattern of bacterial distribution in shipworm hosts follows host species identity and life habits, the presence of abundant GCFs also follows similar patterns. However, as described above, many pathways were found only once or occurred relatively rarely among symbiont genomes and gill metagenomes. In these cases, trends of host symbiont co-occurrence could not be discerned. This observation is reinforced in Fig. 7, where most GCFs in the diagram occur only once (represented by single, unlinked spots). Thus, while the occurrence of common biosynthetic pathways is evolutionarily conserved among host species and thus likely has a uniquely critical role in the symbiosis, most are not conserved. These observations suggest that a more comprehensive sampling of shipworm specimens, species, and cultivated isolates would yield many additional, unanticipated BGCs.

### Variability in conserved shipworm GCFs increases potential compound diversity.

Even among conserved GCFs, variability was observed, as revealed by bulges in the network diagram (Fig. 7). For example, in ubiquitous GCF_3, three different pathway variants are visible. Siderophore pathway GCF_8 contains one central cluster, encoding turnerbactin pathways, and an extended arm that appears to encode compounds related to, but not identical to, turnerbactin.

### Conclusions.

In shipworms, cellulolytic bacteria have long been known to specifically inhabit gills and have been hypothesized to cause an evolutionary path that leads to wood specialization in most of the members of the family, along with drastic morphophysiological modifications (1, 5, 44). These symbionts could be cultivated, although we have only recently been able to sample the full spectrum of major symbionts present in gills. The unexpected finding that T. turnerae T7901 was exceptionally rich in BGCs—i.e., that it was proportionately denser in BGC content than *Streptomyces* spp. (14, 16)—led us to investigate shipworms as a source of new bioactive compounds.

Here, we show that cultivated isolates obtained from shipworm gills accurately represent the bacteria living within the gills. They represent the same species and often are nearly identical at the strain level. They contain many of the same BGCs. The gills of shipworms contain about 1 to 3 major species of symbiotic bacteria, along with a small percentage of other, less consistently occurring bacteria. Complicating this relatively simple picture, there is significant strain variation within shipworms. The observed symbiont species mixtures are representative of the animal lifestyles. For example, K. polythalamius appears to thrive entirely on sulfide oxidation (7), as required in its sediment habitat, while the other shipworms contain various cellulolytic bacteria responsible for wood degradation. D. mannii likely has a more complex lifestyle, since it contains sulfur-oxidizing bacterium strain 2719K and cellulolytic species T. turnerae and strain 2753L.

The key finding is that the BGCs in the metagenomes are represented in the strains in our culture collection. This is a rare event in the biosynthetic literature. In most other marine systems, it has been very challenging to cultivate the symbiotic bacteria responsible for secondary metabolite production (45). In some organisms, such as humans, there are many representative cultivated isolates that produce secondary metabolites, but connecting those metabolites to human biology, or even to their existence in humans, is quite challenging (16, 46). Here, we have defined an experimentally tractable system to investigate chemical ecology that circumvents these limitations. Our results reveal potentially important chemical interactions that would affect a variety of marine ecosystems and a novel and underexplored source of bioactive metabolites for drug discovery.

It has not escaped our notice that this work provides the foundation for understanding the connections between symbiont community composition, secondary metabolite complement, and host lifestyle and ecology. It has proven difficult to link these factors together in relevant models. The existence of methods for aquaculture and transformation for shipworms and their symbiotic bacteria will enable a rigorous, hypothesis-driven understanding of the role of complex metabolism in symbiosis.

## MATERIALS AND METHODS

### Collection and processing of biological material.

Shipworm samples (see Table S1 in the supplemental material) were collected from found wood. Briefly, infested wood was collected and transported immediately to the laboratory or stored in the shade until extraction (<1 day). Specimens were carefully extracted using woodworking tools to avoid damage. Extracted specimens were processed immediately or stored in individual containers of filtered seawater at 4°C until processing. Specimens were checked for viability by siphon retraction in response to stimulation and observation of heartbeat and live specimens selected. Specimens were assigned a unique code, photographed, and identified. Specimens were dissected using a dissecting stereoscope. Taxonomic vouchers (valves, pallets, and siphonal tissue for sequencing host phylogenetic markers) were retained and stored in 70% ethanol. The gill was dissected, rinsed with sterile seawater, and divided for bacterial isolation and metagenomic sequencing. Once the gill was dissected, it was processed immediately or flash-frozen in liquid nitrogen.

All collections followed Nagoya Protocol requirements. Brazilian sampling were performed under SISBIO license number 48388, and genetic resources were accessed under the authorization of the Brazilian National System for the Management of Genetic Heritage and Associated Traditional Knowledge (SisGen permit number A2F0DA0).

Among the animals that we obtained in field collections, we analyzed three specimens each of Bactronophorus thoracites, *Kuphus* spp., Neoteredo reynei, and *Teredo* sp.; two specimens of *Bankia* sp.; and five specimens of Bankia setacea. These animals were divided into three geographical regions (Fig. 1): the Philippines (B. thoracites and D. mannii from Infanta, Quezon, and *Kuphus* spp. from Mindanao and Mabini), Brazil (N. reynei from Rio de Janeiro and *Teredo* sp. and *Bankia* sp. from Ceará), and the United States (B. setacea). The purpose of sampling this range was to determine whether there are any geographical differences in gill symbiont occurrence. Most of the shipworms were obtained from mangrove wood, with the exceptions of B. setacea from unidentified found wood and *Kuphus* spp. from both found wood and mud.

### Bacterial isolation, DNA extraction, and analysis.

Teredinibacter turnerae strains (indicated with “T” prefix) were isolated using the method described previously by Distel el al. (13), while Bankia setacea symbionts (indicated with “Bs” prefix) were obtained using the technique described previously by O’Connor et al. (9). Sulfur-oxidizing symbionts were isolated using the protocol specified previously by Altamia et al. (23). For this study, additional T. turnerae and novel cellulolytic symbionts from Philippine specimens (indicated with “PMS” prefix) were isolated (Table S1). Briefly, dissected gills were homogenized in sterile 75% natural seawater buffered with 20 mM HEPES (pH 8.0) using a Dounce homogenizer. Tissue homogenates were either streaked on shipworm basal medium cellulose (5) plates (1.0% Bacto agar) or stabbed into soft-agar (0.2% Bacto agar) tubes and incubated at 25°C until cellulolytic clearings developed. Cellulolytic bacterial colonies were subjected to several rounds of restreaking to ensure clonal selection. Contents of soft agar tubes with clearings were streaked on fresh cellulose plates to obtain single colonies. Pure colonies were then grown in 6 ml SBM cellulose liquid medium in 16-by-150-mm test tubes until the desired turbidity was observed. For long-term preservation of the isolates, a turbid medium was added to 40% glycerol at a 1:1 ratio and frozen at –80°C. Bacterial cells in the remaining liquid medium were pelleted by centrifugation at 8,000 × *g* and then subjected to genomic DNA isolation. The small-subunit (SSU) ribosomal 16S rRNA gene of the isolates was then PCR amplified using 27F (5′-AGAGTTTGATCCTGGCTCAG-3′) and 1492R (5′-GGTTACCTTGTTACGACTT-3′) from the prepared genomic DNA and sequenced. Phylogenetic analyses of 16S rRNA sequences were performed using programs implemented in Geneious, version 10.2.3. Briefly, sequences were aligned using MAFFT (version 7.388) by using the E-INS-i algorithm. The aligned sequences were trimmed manually, resulting in a final aligned data set of 1,125 nucleotide positions. Phylogenetic analysis was performed using FastTree (version 2.1.11) and the GTR substitution model with optimized Gamma20 likelihood and rate categories per site set to 20.

Genomic DNA used for whole-genome sequencing of novel isolates and select T. turnerae strains was prepared using a cetyltrimethylammonium bromide (CTAB)/phenol-chloroform DNA extraction method detailed elsewhere (https://www.pacb.com/wp-content/uploads/2015/09/DNA-extraction-chlamy-CTAB-JGI.pdf). The purity of the extracted genomic DNA was then assessed spectrophotometrically using Nanodrop, and the quantity was estimated using agarose gel electrophoresis. Samples that passed the quality control steps were submitted to Joint Genome Institute—Department of Energy (JGI-DOE) for whole-genome sequencing. The sequencing platform and assembly method used to generate the final isolate genome sequences used in this study are detailed in Table S1A.

### Metagenomic DNA extraction.

Gill tissue samples from Philippine shipworm specimens (Table S1B) were flash-frozen in liquid nitrogen and stored at –80°C prior to processing. Bulk gill genomic DNA was purified by the use of a Qiagen blood and tissue genomic DNA kit using the manufacturer’s suggested protocol.

Gill tissue samples from Brazil shipworm specimens were pulverized by flash-freezing in liquid nitrogen and submitted to metagenomic DNA purification by adapting a protocol previously optimized for total DNA extraction from cnidaria tissues (47, 48). Briefly, shipworms gills were carefully dissected (with care taken not to include contamination from other organs), submitted to a series of five washes with 3:1 sterile seawater/distilled water for removal of external contaminants, and macerated until they were powdered in liquid nitrogen. Powdered tissues (∼150 mg) were then transferred to 2-ml microtubes containing 1 ml of lysis buffer (2% [w/v] CTAB [Sigma-Aldrich], 1.4 M NaCl, 20 mM EDTA, 100 mM Tris-HCl [pH 8.0], with freshly added 5 μg proteinase K [vol/vol] [Invitrogen] and 1% 2-mercaptoethanol [Sigma-Aldrich]) and submitted to five freeze-thawing cycles (–80°C to 65°C). Proteins were extracted by washing twice with phenol-chloroform-isoamyl alcohol (25:24:1) and once with chloroform. Metagenomic DNA was precipitated with isopropanol and 5 M ammonium acetate, washed with 70% ethanol, and eluted in TE buffer (10 mM Tris-HCl, 1 mM EDTA). Metagenomic libraries were prepared using a Nextera XT DNA sample preparation kit (Illumina) and sequenced with 600-cycle MiSeq reagent kit chemistry (v3; Illumina) (300-bp paired-end runs) using a MiSeq desktop sequencer.

### Metagenome sequencing and assembly.

Five Bankia setacea metagenome sequencing raw read files were obtained from the JGI database and reassembled using the methods described below (for accession numbers, see Table S1A). Kuphus polythalamius gill metagenomes (KP2132G and KP2133G) were obtained from a previous study (7). Metagenomes from *Kuphus* sp. specimen KP3700G and Dicyathifer mannii and Bactronophorus thoracites specimens were sequenced using an Illumina HiSeq 2000 sequencer with ∼350-bp insertions and 125-bp paired-end runs at the Huntsman Cancer Institute’s High-Throughput Genomics Center at the University of Utah. Illumina fastq reads were trimmed using Sickle (Version 1.33) (49) with the following parameters: pe sanger -q 30 –l 125. The trimmed FASTQ files were converted to FASTA files and merged using the Perl script “fq2fq” in IBDA_ud (version 2) package (50). Merged FASTA files were assembled using IDBA_ud (version 2) with standard parameters in the Center for High Performance Computing at the University of Utah. For the metagenome samples from Brazil, all Neoteredo reynei gill metagenomic samples previously analyzed were resequenced here to improve coverage depth (27). *Teredo* sp. and *Bankia* sp. gill metagenomes were sequenced using Illumina MiSeq. The raw reads were assembled using either the metaspades pipeline of SPAdes (version 3.11.1) (51, 52) or IDBA-UD (version 2) (50). Before assembly, raw reads were merged using BBMerge (v9.02) (53). Nonmerged reads were filtered and trimmed using FaQCs (Version 1.34) (54).

### Identification of bacterial sequences in metagenomic data.

Assembly-assisted binning was used to sort and analyze trimmed reads and the contigs were assembled into clusters putatively representing single genomes using MetaAnnotator beta version (55). Each binned genome was retrieved using SAMtools (version: 1.10) (56, 57). To identify bacterial genomes, genes for each bin were identified with Prodigal (58). Protein sequences for bins with coding density levels of >50% were searched against the NCBI nr database with DIAMOND (v0.9.32) (59). Bins with 60% of the genes hitting the bacterial subject in the nr database were considered to have originated from bacteria.

For the B, setacea metagenome samples and the ones from Brazil, structural and functional annotations were carried out using DFAST (v1.1.5) (60), including only contigs with lengths of ≥500 bp. All metagenomes were binned using Autometa (version 2019) (61). First, the taxonomic identity of each contig was predicted using make_taxonomy_table.py, including only contigs that were ≥1,000 bp in length. Predicted bacterial and archaeal contigs were binned (with recruitment performed via supervised machine learning) using run_autometa.py. The statistics of the symbiont bins was generated by CheckM (62).

### gANI comparisons and calculation of read counts.

Each bacterial bin was compared to the 23 shipworm isolate genomes using gANI and AF values (63). With a cutoff AF value of >0.5 and gANI value of >0.9, the bacterial bins from each metagenome were mapped to cultivated bacterial genomes and the cultivated bacterial genomes mapped against each other (Table S2). The major but not mapped bins in each genome were classified using gtdb-tk (version 1.1.1) (64). The read counts for each mapped bin were either retrieved from the output of MetaAnnotator (beta version) or calculated using bbwrap.sh (https://sourceforge.net/projects/bbmap/) with the following parameters: kfilter = 22 subfilter = 15 maxindel = 80.

### Building BGC similarity networks.

BGCs were predicted from the bacterial contigs of each metagenome and from cultivated bacterial genomes using antiSMASH 4.0 (31) (see Fig. S6 in the supplemental material). From the predictions, only the BGCs for PKSs, NRPSs, siderophores, terpenes, homoserine lactones, and thiopeptides (as well as combinations of these biosynthetic enzyme families) were included in the succeeding analyses. An all-versus-all comparison of these BGCs was performed using MultiGeneBlast (v1.1.14) (32) and a previously reported protocol (65). The bidirectional MultiGeneBlast BGC-to-BGC hits were considered to be reliable. In the metagenome data, some truncated BGCs showed only single-directional correlation to a full-length BGC. Those single-directional hits were refined as follows: protein translations of all coding sequences from the BGCs were compared in an all-versus-all fashion using blastp search. Only those protein hits that had at least 60% identity to and at least 80% coverage of both query and subject were considered to represent valid hits. Single-directional MultiGeneBlast BGC-to-BGC hits were retained if the number of proteins represented at least *n* − 2 (*n* is the number of proteins in the truncated BGC) passing the blastp refining. The remaining MultiGeneBlast hits were used to construct a network in Cytoscape (v3.7.0) (66). Finally, each BGC cluster (GCF) that had a relatively low number of bidirectional correlations was manually checked by examining the MultiGeneBlast alignment.

10.1128/mSystems.00261-20.6 FIG S6 Raw antiSMASH output, showing total BGCs in shipworm isolates. Download FIG S6, EPS file, 0.5 MB. Copyright © 2020 Altamia et al. 2020 Altamia et al. This content is distributed under the terms of the Creative Commons Attribution 4.0 International license.

### Occurrence of GCFs in metagenomes.

On the basis of the GCFs identified in previous step, the core biosynthetic proteins from each GCF were extracted and queried (NCBI tblastn) against each metagenome assembly. Thresholds of query coverage of >50% and identity of >90% were applied to remove the nonspecific hits, and the remaining hits, in combination with the MultiGeneBlast hits, were used to make the matrix of occurrences of GCFs in metagenomes.

### Data availability.

The raw sequencing data are available in GenBank under accession numbers SRX7665675, SRX7665685, SRX7665686, SRX7665676, SRX7665684, SRX7665687, SRX7665688, SRX7665689, SRX7665690, SRX7665691, SRX7665677, SRX7665678, SRX7665679, SRX7665680, SRX7665681, SRX7665682, and SRX7665683 or in JGI (https://img.jgi.doe.gov/cgi-bin/m/main.cgi?) under IMG Genome identifiers 3300000111, 3300000024, 3300000110, 3300000107, 2070309010, 2541046951, 2510917000, 2513237135, 2513237099, 2519899652, 2519899664, 2519899663, 2524614873, 2523533596, 2540341229, 2571042908, 2579779156, 2558309032, 2541046951, 2545555829, 2767802764, 2531839719, 2528768159, 2503982003, 2524614822, 2574179784, 2751185674, 2574179721, and 2751185671. For details, please see Table S1.
