# Correction for Altamia et al., “Secondary Metabolism in the Gill Microbiota of Shipworms (Teredinidae) as Revealed by Comparison of Metagenomes and Nearly Complete Symbiont Genomes”

**DOI:** 10.1128/mSystems.00288-21

**Published:** 2021-04-13

**Authors:** Marvin A. Altamia, Zhenjian Lin, Amaro E. Trindade-Silva, Iris Diana Uy, J. Reuben Shipway, Diego Veras Wilke, Gisela P. Concepcion, Daniel L. Distel, Eric W. Schmidt, Margo G. Haygood

**Affiliations:** a Ocean Genome Legacy Center, Department of Marine and Environmental Science, Northeastern University, Nahant, Massachusetts, USA; b The Marine Science Institute, University of the Philippines Diliman, Quezon City, Philippines; c Department of Medicinal Chemistry, University of Utah, Salt Lake City, Utah, USA; d Bioinformatic and Microbial Ecology Laboratory—BIOME, Federal University of Bahia, Salvador, Bahia, Brazil; e Drug Research and Development Center, Department of Physiology and Pharmacology, Federal University of Ceará, Ceará, Brazil; f Philippine Genome Center, University of the Philippines Diliman, Quezon City, Philippines; g Institute of Marine Science, School of Biological Sciences, University of Portsmouth, Portsmouth, United Kingdom

## AUTHOR CORRECTION

Volume 5, no. 3, e00261-20, 2020, https://doi.org/10.1128/mSystems.00261-20. Page 16: The following should be added to the end of the third paragraph of Acknowledgments. “Biosynthetic analysis was also supported by NIH R35GM122521.”

