# Supplementary material for: Secondary Metabolism in the Gill Microbiota of Shipworms (Teredinidae) as Revealed by Comparison of Metagenomes and Nearly Complete Symbiont Genomes
Source: mSystems. 2020 Jun 30;5(3):e00261-20. doi: 10.1128/mSystems.00261-20 (PMC7329324; doi:10.1128/mSystems.00261-20)
Supplement: TABLE S1 [file mSystems.00261-20-st001.docx]

**A. Shipworm gill metagenomes used in this study.**

| **#** | **Gill metagenome** | **PMS-ICBG sample codes** | **Source shipworm species** | **Location** | **Coordinates** | **Sequencing center** | **Sequencing platform** | **Assembler** | **Reads, post trim** | **Size in bp** | **No. of contigs** | **N50** | **%GC** | **IMG Genome ID** | **SRA accession #** |
| --- | --- | --- | --- | --- | --- | --- | --- | --- | --- | --- | --- | --- | --- | --- | --- |
| 1 | DM2722G | PMS-2722P | *Dicyathifer mannii* specimen PMS-2717Y | Infanta, Quezon, Philippines | N 14.68367°, E 121.63690° | Huntsman Cancer Institute, University of Utah | Illumina HiSeq 2000 | IDBA_ud | 187291588 | 1235295176 | 924064 | 2095 | 34.9 |  | SRX7665675 |
| 2 | BT2771G | PMS-2771X | *Bactronophorus thoracites* specimen PMS-2769U | Infanta, Quezon, Philippines | N 14.68367°, E 121.63690° | Huntsman Cancer Institute, University of Utah | Illumina HiSeq 2000 | IDBA_ud | 177392546 | 1056707310 | 813604 | 2023 | 35.4 |  | SRX7665685 |
| 3 | BT2849G | PMS-2849Y | *Bactronophorus thoracites* specimen PMS-2839H | Infanta, Quezon, Philippines | N 14.68367°, E 121.63690° | Huntsman Cancer Institute, University of Utah | Illumina HiSeq 2000 | IDBA_ud | 193099534 | 1059162705 | 814617 | 2024 | 35.4 |  | SRX7665686 |
| 4 | DM2858G | PMS-2858W | *Dicyathifer mannii* specimen PMS-2823T | Infanta, Quezon, Philippines | N 14.68367°, E 121.63690° | Huntsman Cancer Institute, University of Utah | Illumina HiSeq 2000 | IDBA_ud | 186697500 | 1236681788 | 928980 | 2083 | 34.9 |  | SRX7665676 |
| 5 | DM3770G | PMS-3770U | *Dicyathifer mannii* specimen PMS-3768S | Infanta, Quezon, Philippines | N 14.68367°, E 121.63690° | Huntsman Cancer Institute, University of Utah | Illumina HiSeq 2000 | IDBA_ud | 297553066 | 1328488478 | 1067922 | 1946 | 34.9 |  | SRX7665684 |
| 6 | BT3790G | PMS-3790S | *Bactronophorus thoracites* specimen PMS-3779S | Infanta, Quezon, Philippines | N 14.68367°, E 121.63690° | Huntsman Cancer Institute, University of Utah | Illumina HiSeq 2000 | IDBA_ud | 309554332 | 1127330840 | 927279 | 1873 | 35.4 |  | SRX7665687 |
| 10 | KP3700G | PMS-3700M | *Kuphus polythalamius* specimen PMS-3696Y (wood-boring) | Mabini, Batangas, Philippines | N 13.75843°, E 120.92586° | Huntsman Cancer Institute, University of Utah | Illumina HiSeq 2000 | IDBA_ud | 82015762 | 734092095 | 358482 | 4300 | 37.6 |  | SRX7665688 |
| 11 | KP2132G | PMS-2246K and PMS-2249P | *Kuphus polythalamius* specimen PMS-2132W (mud-dwelling) | Kalamansig, Sultan Kudarat, Philippines | N 6.53631°, E 124.048365° | Huntsman Cancer Institute, University of Utah | Illumina HiSeq 2000 | IDBA_ud | 318294870 | 772720664 | 424816 | 4530 | 37.6 |  | SRX7665689 |
| 12 | KP2133G | PMS-2157H, PMS-2116M, and PMS-2110W | *Kuphus polythalamius* specimen PMS-2133X (mud-dwelling) | Kalamansig, Sultan Kudarat, Philippines | N 6.53631°, E 124.048365° | Huntsman Cancer Institute, University of Utah | Illumina HiSeq 2000 | IDBA_ud | 329174268 | 795400237 | 500141 | 3879 | 37.4 |  | SRX7665690 |
| 13 | BSG1 | - | *Bankia setacea* | Puget Sound, Washington, USA | N 47.85072°, W 122.33843° | Joint Genome Institute -Department of Energy | Illumina HiSeq 2000 | SOAPdenovo, Newbler, and Minimus2 | 154360930 | 563042012 | 761912 | 985 | 35.0 | 3300000111 | - |
| 14 | BSG3 | - | *Bankia setacea* | Puget Sound, Washington, USA | N 47.957498°, W 122.529373° | Joint Genome Institute -Department of Energy | Illumina HiSeq 2000 | SOAPdenovo, Newbler, and Minimus2 | 144540774 | 620222960 | 648493 | 1550 | 34.9 | 3300000024 | - |
| 15 | BSG2 | - | *Bankia setacea* | Puget Sound, Washington, USA | N 47.957498°, W 122.529373° | Joint Genome Institute -Department of Energy | Illumina HiSeq 2000 | SOAPdenovo, Newbler, and Minimus2 | 180149584‬ | 540217764 | 793976 | 860 | 34.8 | 3300000110 | - |
| 17 | BSG4 | - | *Bankia setacea* | Puget Sound, Washington, USA | N 47.85072°, W 122.33843° | Joint Genome Institute -Department of Energy | Illumina HiSeq 2000 | SOAPdenovo, Newbler, and Minimus2 | 159707004‬ | 574332630 | 692986 | 1194 | 34.6 | 3300000107 | - |
| 19 | BS_sunk | - | *Bankia setacea* | Puget Sound, Washington, USA | N 47.85072°, W 122.33843 | Joint Genome Institute -Department of Energy | Illumina, 454 GS FLX Titanium | Newbler and Velvet | - | 26539887 | 38227 | 1943 | 45.2 | 2070309010 | - |
| 20 | NR01 | - | *Neoteredo reynei* | Coroa grande Mangrove - Sepetiba bay, Rio de Janeiro State, BR | 22.9081670° S 43.8756390° W | CEGENBIO | Illumina MiSeq | SPAdes | 9224156 | 313630826 | 413893 | 779 | 37.3 | - | SRX7665691 |
| 21 | NR02 | - | *Neoteredo reynei* | Coroa grande Mangrove - Sepetiba bay, Rio de Janeiro State, BR | 22.9081670° S 43.8756390° W | CEGENBIO | Illumina MiSeq | SPAdes | 18338062 | 416566737 | 468503 | 986 | 37.2 | - | SRX7665677 |
| 22 | NR03 | - | *Neoteredo reynei* | Coroa grande Mangrove - Sepetiba bay, Rio de Janeiro State, BR | 22.9081670° S 43.8756390° W | CEGENBIO | Illumina MiSeq | SPAdes | 13078802 | 309408486 | 414159 | 769 | 38.2 | - | SRX7665678 |
| 23 | TBF02 | - | *Teredo* sp. | Environmental Preservation Area of Pacoti river, Ceará State, Brazil | S 3.843111, W 38.422695 (3°50'35.2"S 38°25'21.7"W) | CEGENBIO | Illumina MiSeq | SPAdes | 3565711 | 74108472 | 236018 | 1037 | 40.1 |  | SRX7665679 |
| 24 | TBF03 | - | *Bankia* sp. | Environmental Preservation Area of Pacoti river, Ceará State, Brazil | S 3.843111, W 38.422695 (3°50'35.2"S 38°25'21.7"W) | CEGENBIO | Illumina MiSeq | SPAdes | 2205607 | 33524312 | 75538 | 1123 | 41.2 |  | SRX7665680 |
| 25 | TBF05 | - | *Bankia* sp. | Environmental Preservation Area of Pacoti river, Ceará State, Brazil | S 3.843111, W 38.422695 (3°50'35.2"S 38°25'21.7"W) | CEGENBIO | Illumina MiSeq | SPAdes | 3632367 | 107179837 | 230494 | 995 | 37.4 |  | SRX7665681 |
| 26 | TBF07 | - | *Teredo* sp. | Environmental Preservation Area of Pacoti river, Ceará State, Brazil | S 3.843111, W 38.422695 (3°50'35.2"S 38°25'21.7"W) | CEGENBIO | Illumina MiSeq | SPAdes | 3731031 | 78684542 | 258368 | 965 | 38.6 |  | SRX7665682 |
| 27 | TBF09 | - | *Teredo* sp. | Environmental Preservation Area of Pacoti river, Ceará State, Brazil | S 3.843111, W 38.422695 (3°50'35.2"S 38°25'21.7"W) | CEGENBIO | Illumina MiSeq | SPAdes | 4029653 | 108441874 | 340072 | 948 | 38.1 |  | SRX7665683 |

**B: Shipworm symbiont genomes.**

| **#** | **Code in the manuscript** | **Isolate name** | **Metabolic type** | **Host shipworm** | **Location** | **Coordinates** | **Sequencing center** | **Sequencing platform** | **Sequence assembler** | **Estimated genome size** | **No. of contigs/scaffolds** | **N50** | **%GC** | **IMG Genome ID** |
| --- | --- | --- | --- | --- | --- | --- | --- | --- | --- | --- | --- | --- | --- | --- |
| 1 | T7901 | *T. turnerae* strain T7901 | Cellulolytic | *Bankia gouldi* | Beaufort, North Carolina USA | N 34.71737°, W 76.67198° | J. Craig Venter Institute | 454, Sanger | Celera Assembler and custom software | 5,193,164 | 1 (closed circular) | Not applicable | 50.89 | 2541046951 |
| 2 | T8415 | *T. turnerae* strain T8415 | Cellulolytic | *Bankia gouldi* | Fort Pierce, Florida, USA | N 27.48063°, W 80.30967° | JGI-DOE | Illumina | ALLPATHS | 5,158,349 | 50 | Scaffold N/L50: 5/398.1 Kbp  Contig N/L50: 6/395.4 kbp | 50.78 | 2510917000 |
| 3 | T8602 | *T. turnerae* strain T8602 | Cellulolytic | *Dicyathifer mannii* | Townsville, Queensland, Australia | S 19.27631°, E 147.05784° | JGI-DOE | Illumina | ALLPATHS | 5,097,488 | 59 | Scaffold N/L50: 6/291.7 kbp  Contig N/L50: 2/291.7 kbp | 51.03 | 2513237135 |
| 4 | T7902 | *T. turnerae* strain T7902 | Cellulolytic | *Lyrodus pedicellatus* | Long Beach, California, USA | N 33.76138°, W 118.17281° | JGI-DOE | Illumina | ALLPATHS | 5,387,817 | 72 | Scaffold N/L50: 11/176.4 kbp  Contig N/L50: 11/176.4 kbp | 50.81 | 2513237099 |
| 5 | T8402 | *T. turnerae* strain T8402 | Cellulolytic | *Teredora malleolus* | Floating wood in the Atlantic Ocean | N 38.30667°, W 69.59333° | JGI-DOE | Illumina | Velvet (1.1.04) and ALLPATHS-LG | 5,166,130 | 27 | Scaffold N/L50: 6/348.4 kbp  Contig N/L50: 7/315.4 kbp | 50.86 | 2519899652 |
| 6 | T8412 | *T. turnerae* strain T8412 | Cellulolytic | *Lyrodus bipartitus* | Jim Island, Fort Piece, Florida, USA | N 27.476944°, W 80.311944° | JGI-DOE | Illumina | Velvet (1.1.04) and ALLPATHS-LG | 5,147,360 | 58 | Scaffold N/L50: 10/205.3 kbp  Contig N/L50: 10/205.3 kbp | 51.07 | 2519899664 |
| 7 | T0609 | *T. turnerae* strain T0609 | Cellulolytic | *Lyrodus pedicellatus* | Long Beach, California, USA | N 33.76138°, W 118.17281° | JGI-DOE | Illumina | Velvet (1.1.04) and ALLPATHS-LG | 5,069,061 | 49 | Scaffold N/L50: 7/246.6 kbp  Contig N/L50: 7/246.6 kbp | 51.15 | 2519899663 |
| 8 | 991H | *T. turnerae* strain PMS-991H.S.0a.06 | Cellulolytic | *Lyrodus pedicellatus* specimen PMS-988W | Panglao, Bohol, Philippines | N 9.54558°, E 123.76030° | JGI-DOE | Illumina | ALLPATHS-LG | 5,279,031 | 13 | Scaffold N/L50: 2/1.8 Mbp  Contig N/L50: 3/888.4 kbp | 51.07 | 2524614873 |
| 9 | T8513 | *T. turnerae* strain T8513 | Cellulolytic | *Teredo navalis* | São Paulo, Brazil | S 23.81992°, W 45.40517° | JGI-DOE | Illumina | Velvet (1.1.04) and ALLPATHS-LG | 5,268,281 | 84 | Scaffold N/L50: 9/189.8 kbp  Contig: 8/189.8 kbp | 50.92 | 2523533596 |
| 10 | 1133Y | *T. turnerae* strain PMS-1133Y.S.0a.04 | Cellulolytic | *Lyrodus* sp. specimen PMS-1128S | Panglao, Bohol, Philippines | N 9.59670°, E 123.74990° | JGI-DOE | Illumina | ALLPATHS-LG | 5,134,977 | 6 | Scaffold N/L50: 1/3.2 Mbp  Contig N/L50: 4/607.0 kbp | 50.85 | 2540341229 |
| 11 | 1675L | *T. turnerae* strain PMS-1675L.S.0a.01 | Cellulolytic | *Kuphus polythalamius* specimen PMS-1672Y | Kalamansig, Sultan Kudarat, Philippines | N 6.53631°, E 124.04836° | JGI-DOE | PacBio | HGAP 2.1.1 | 5,283,781 | 1 (closed circular) | Not applicable | 51.05 | 2571042908 |
| 12 | 2753L | PMS-27553L.S.0a.02 | Cellulolytic | *Bactronophorus thoracites* specimen PMS-2749X | Infanta, Quezon, Philippines | N 14.68367°, E 121.63690° | JGI-DOE | PacBio | HGAP 2.1.1 | 6,056,039 | 2 | Scaffold N/L50: 1/4.4 Mbp | 47.96 | 2579779156 |
| 13 | 1120W | PMS-1120W.S.0a.04 | Cellulolytic | *Teredo fulleri* specimen PMS-1114L | Panglao, Bohol, Philippines | N 9.59670°, E 123.74990° | JGI-DOE | PacBio | HGAP 2.0.1 | 5,699,307 | 1 (closed circular) | Not applicable | 50.39 | 2558309032 |
| 14 | 2052S | PMS-2052S.S.stab0a.01 | Cellulolytic | *Bactronophorus thoracites* specimen PMS-1959H | Butuan, Agusan del Norte, Philippines | N 8.98650°, E 125.45768° | JGI-DOE | Illumina | ALLPATHS -LG | 5,635,926 | 3 | Scaffold N/L50: 1/5.6 Mbp  Contig: 3/981.6 kbp | 54.68 | 2541046951 |
| 15 | BS12 | BS12 | Cellulolytic | *Bankia setacea* | Puget Sound, Washington, USA | N 47.95749°, W 122.52937° | JGI-DOE | PacBio | HGAP 2.0.0 | 4,921,245 | 3 | Contig: 1/4.7 Mbp | 45.72 | 2545555829 |
| 16 | BS08 | BS08 | Cellulolytic | *Bankia setacea* | Puget Sound, Washington, USA | N 47.95749°, W 122.52937° | JGI-DOE | Illumina | Velvet v. DEC-2010 | 4,814,259 | 90 | Scaffold N/L50: 7/255.3 Mbp  Contig: 14/112.2 kbp | 47.18 | 2767802764 |
| 17 | BSC2 | BSC2 | Cellulolytic | *Bankia setacea* | Puget Sound, Washington, USA | N 47.95749°, W 122.52937° | New England Biolabs | PacBio | HGAP 2.0.1 | 5,414,953 | 10 | 4.2 Mbp | 47.31 | 2531839719 |
| 18 | BS31 | BS31 | Cellulolytic | *Bankia setacea* | Puget Sound, Washington, USA | N 47.95749°, W 122.52937° | JGI-DOE | PacBio | Velvet 1.1.04 and ALLPATHS-LG | 5,017,353 | 46 | Scaffold N/L50: 5/341.1 kbp  Contig: 8/260.1 kbp | 47.60 | 2528768159 |
| 19 | BS02 | Teredinibacter waterburyi | Cellulolytic | *Bankia setacea* | Puget Sound, Washington, USA | N 47.95749°, W 122.52937° | JGI-DOE | Illumina | Velvet v. DEC-2010 | 3,886,134 | 141 | Contig: 8/176.2 kbp | 47.76 | 2503982003 |
| 20 | 1162T | PMS-1162T.S.0a.05 | Cellulolytic | *Lyrodus* sp. specimen PMS-1157K | Talibon, Bohol, Philippines | N 10.30748°, E 124.40168° | JGI-DOE | Illumina and PacBio | ALLPATHS-LG | 4,404,964 | 1 (closed circular) | Not applicable | 47.72 | 2524614822 |
| 21 | 1081L | PMS-1081L.S.0a.03 | Agarolytic | *Bankia* sp. specimen PMS-1083P | Panglao, Bohol, Philippines | N 9.59670°, E 123.74990° | JGI-DOE | PacBio | HGAP 2.1.1 | 4,255,513 | 13 | Scaffold N/L50: 568.3 kbp | 53.67 | 2574179784 |
| 22 | 2141T | *Thiosocius teredinicola* PMS-2141T.STBD.0c.01a | Sulfur-oxidizing | *Kuphus polythalamius* specimen PMS-2133X | Kalamansig, Sultan Kudarat, Philippines | N 6.53631°, E 124.048365° | JGI-DOE | PacBio | HGAP 2.0.1 | 4,790,451 | 1 (closed circular) | Not applicable | 60.08 | 2751185674 |
| 23 | 2719K | *Thiosocius* sp. PMS-2719K.STB50.0a.01 | Sulfur-oxidizing | *Dicyathifer mannii* specimen PMS-2715W | Infanta, Quezon, Philippines | N 14.68367°, E 121.63690° | JGI-DOE | PacBio | HGAP 2.0.1 | 5,077,565 | 1 (closed circular) | Not applicable | 58.55 | 2574179721 |
| 24 | Ga0198945 | *Agarilytica rhodophyticola* strain 017 | Agarolytic | Associated with the seaweed *Gracilaria blodgettii* | Lingshui County, Hainan, China | N 18.40828° , E 110.0623° | JGI-DOE | Illumina and PacBio | SOAPdenovo 2.04; Celera Assembler 8.0 | 6,878,829 | 1 (closed circular) | Not applicable | 40.97 | 2751185671 |

**C: Statistics of genomic bins from metagenomes generated by checkM.**

| Bin Id | Completeness | Contamination | Strain heterogeneity |
| --- | --- | --- | --- |
| BSG1_1_1 | 97.55 | 8.42 | 44.44 |
| BSG2_2_0 | 95.72 | 0.92 | 40 |
| BSG2_2_1 | 60.34 | 0 | 0 |
| BSG2_2_4 | 14.35 | 0 | 0 |
| BSG2_2_9 | 2.16 | 0 | 0 |
| BSG3_2_0 | 93.8 | 7.74 | 25.53 |
| BSG4_1_0 | 96 | 2.96 | 7.69 |
| BT2771G_1251 | 0 | 0 | 0 |
| BT2771G_1266 | 56.91 | 0.54 | 100 |
| BT2771G_2629 | 41.87 | 1.05 | 20 |
| BT2849G_1158 | 12.07 | 0 | 0 |
| BT2849G_1418 | 0 | 0 | 0 |
| BT2849G_1523 | 0 | 0 | 0 |
| BT2849G_1577 | 0 | 0 | 0 |
| BT2849G_1909 | 85 | 0.97 | 25 |
| BT2849G_2869 | 0 | 0 | 0 |
| BT3790G_1208 | 0 | 0 | 0 |
| BT3790G_1493 | 3.45 | 0 | 0 |
| BT3790G_1981 | 0 | 0 | 0 |
| BT3790G_2237 | 82.03 | 2.78 | 78.57 |
| BT3790G_3135 | 0 | 0 | 0 |
| DM2722G_1447 | 43.86 | 3.51 | 100 |
| DM2722G_1691 | 58.62 | 0 | 0 |
| DM2722G_1870 | 34.03 | 5.05 | 81.82 |
| DM2722G_3144 | 31.58 | 3.32 | 64.71 |
| DM2722G_497 | 12.5 | 4.17 | 0 |
| DM2722G_579 | 95.41 | 2.96 | 72.22 |
| DM2858G_1105 | 3.45 | 0 | 0 |
| DM2858G_1458 | 93.8 | 3.08 | 68.18 |
| DM2858G_2488 | 0 | 0 | 0 |
| DM2858G_2501 | 8.33 | 0 | 0 |
| DM2858G_2907 | 73.68 | 15.95 | 80.77 |
| DM2858G_3045 | 41.38 | 1.72 | 100 |
| DM2858G_3735 | 57.5 | 1 | 50 |
| DM3770G_1109 | 0 | 0 | 0 |
| DM3770G_1432 | 0 | 0 | 0 |
| DM3770G_2006 | 67.33 | 11.49 | 79.63 |
| DM3770G_2725 | 95.19 | 11.27 | 89.61 |
| DM3770G_2751 | 0 | 0 | 0 |
| DM3770G_2901 | 0 | 0 | 0 |
| DM3770G_2983 | 15.3 | 2.81 | 61.54 |
| DM3770G_3242 | 6.5 | 2.59 | 50 |
| DM3770G_3460 | 0 | 0 | 0 |
| DM3770G_5 | 7.76 | 0 | 0 |
| DM3770G_615 | 6.9 | 0 | 0 |
| DM3770G_994 | 8.62 | 3.45 | 33.33 |
| KP2132G_2024 | 0 | 0 | 0 |
| KP2132G_487 | 30.02 | 27.62 | 96.15 |
| KP2132G_543 | 72.26 | 81.97 | 90.48 |
| KP2132G_930 | 0 | 0 | 0 |
| KP2133G_110 | 0 | 0 | 0 |
| KP2133G_12 | 11.31 | 4.77 | 100 |
| KP2133G_1401 | 0 | 0 | 0 |
| KP2133G_1537 | 1.72 | 1.72 | 100 |
| KP2133G_1802 | 0 | 0 | 0 |
| KP2133G_407 | 0 | 0 | 0 |
| KP2133G_561 | 0 | 0 | 0 |
| KP2133G_574 | 56.92 | 29.8 | 90 |
| KP2133G_581 | 0 | 0 | 0 |
| KP2133G_742 | 0 | 0 | 0 |
| KP3700G_1264 | 75.49 | 48.66 | 88.98 |
| KP3700G_1558 | 17.38 | 13.06 | 85.71 |
| KP3700G_2285 | 1.72 | 1.72 | 100 |
| KP3700G_254 | 0 | 0 | 0 |
| NR01_83_0 | 79.46 | 50.71 | 56.8 |
| NR01_uc | 56.07 | 6.08 | 88.89 |
| NR02_1_1 | 98.35 | 5.46 | 58.33 |
| NR03_1_5 | 0.42 | 0 | 0 |
| NR03_3_0 | 29.22 | 6.53 | 74.19 |
| NR03_uc | 71.19 | 34.83 | 62.96 |
| TBF02_1_1 | 21.92 | 0.03 | 0 |
| TBF02_3_0 | 67.56 | 7.37 | 18.75 |
| TBF03_2_0 | 77.1 | 7.36 | 9.68 |
| TBF05_2_0 | 94.73 | 3.41 | 15.79 |
| TBF05_uc | 3.63 | 0.02 | 100 |
| TBF07_1_1 | 30.1 | 0.05 | 50 |
| TBF09_17_0 | 38.04 | 0 | 0 |
| TBF09_2_0 | 15.32 | 0.09 | 50 |
| T0609 | 100 | 0.52 | 0 |
| T7902 | 100 | 0 | 0 |
| T8402 | 100 | 0 | 0 |
| T8412 | 100 | 0.72 | 0 |
| T8415 | 100 | 0.52 | 0 |
| T8513 | 100 | 0.59 | 0 |
| T8602 | 100 | 0.11 | 0 |
| 1133Y | 100 | 0.44 | 0 |
| 1162T | 99.5 | 0.86 | 0 |
| 1675L | 100 | 0.14 | 0 |
| 2141T | 99.94 | 1.31 | 0 |
| 2719K | 99.94 | 1.99 | 0 |
| 2753L | 100 | 1.56 | 0 |
| 991H | 100 | 0.01 | 0 |
| BS08 | 99.46 | 0.58 | 0 |
