# Supplementary material for: Secondary Metabolism in the Gill Microbiota of Shipworms (Teredinidae) as Revealed by Comparison of Metagenomes and Nearly Complete Symbiont Genomes
Source: mSystems. 2020 Jun 30;5(3):e00261-20. doi: 10.1128/mSystems.00261-20 (PMC7329324; doi:10.1128/mSystems.00261-20)
Supplement: TABLE S2 [file mSystems.00261-20-st002.docx]

| genome_ID1 | genome_ID2 | AF | gANI | edge_score=AF*gANI | genome_length |
| --- | --- | --- | --- | --- | --- |
| 1133Y | 1675L | 0.88837 | 0.97601 | 0.867058 | 4416264 |
| 1133Y | 991H | 0.917913 | 0.976538 | 0.896377 | 4416264 |
| 1133Y | NR01_83_0.fasta | 0.625963 | 0.931249 | 0.582927 | 4416264 |
| 1133Y | NR02_1_1.fasta | 0.828551 | 0.924503 | 0.765998 | 4416264 |
| 1133Y | NR03_uc.fasta | 0.627183 | 0.936548 | 0.587387 | 4416264 |
| 1133Y | T0609 | 0.880447 | 0.974621 | 0.858102 | 4416264 |
| 1133Y | T7901 | 0.901882 | 0.930208 | 0.838938 | 4416264 |
| 1133Y | T7902 | 0.892839 | 0.976096 | 0.871497 | 4416264 |
| 1133Y | T8402 | 0.89629 | 0.931583 | 0.834969 | 4416264 |
| 1133Y | T8412 | 0.896951 | 0.972772 | 0.872529 | 4416264 |
| 1133Y | T8415 | 0.890276 | 0.932962 | 0.830594 | 4416264 |
| 1133Y | T8513 | 0.903741 | 0.975097 | 0.881235 | 4416264 |
| 1133Y | T8602 | 0.897988 | 0.930602 | 0.835669 | 4416264 |
| 1133Y | TBF05_2_0.fasta | 0.818783 | 0.968715 | 0.793167 | 4416264 |
| 1675L | 1133Y | 0.872475 | 0.975545 | 0.851139 | 4536378 |
| 1675L | 991H | 0.890956 | 0.98154 | 0.874509 | 4536378 |
| 1675L | NR01_83_0.fasta | 0.633585 | 0.931447 | 0.590151 | 4536378 |
| 1675L | NR02_1_1.fasta | 0.827928 | 0.919958 | 0.761659 | 4536378 |
| 1675L | NR03_uc.fasta | 0.635214 | 0.935692 | 0.594365 | 4536378 |
| 1675L | T0609 | 0.887587 | 0.979341 | 0.86925 | 4536378 |
| 1675L | T7901 | 0.868763 | 0.923789 | 0.802554 | 4536378 |
| 1675L | T7902 | 0.905002 | 0.986385 | 0.89268 | 4536378 |
| 1675L | T8402 | 0.86934 | 0.925605 | 0.804665 | 4536378 |
| 1675L | T8412 | 0.877281 | 0.979048 | 0.8589 | 4536378 |
| 1675L | T8415 | 0.894459 | 0.924962 | 0.827341 | 4536378 |
| 1675L | T8513 | 0.892232 | 0.982003 | 0.876175 | 4536378 |
| 1675L | T8602 | 0.855345 | 0.924807 | 0.791029 | 4536378 |
| 1675L | TBF05_2_0.fasta | 0.783679 | 0.97568 | 0.76462 | 4536378 |
| 2141T | KP2132G_543 | 0.667543 | 0.904162 | 0.603567 | 4217910 |
| 2719K | DM2722G_579 | 0.871057 | 0.994387 | 0.866168 | 4518363 |
| 2719K | DM2858G_1458 | 0.855756 | 0.991247 | 0.848266 | 4518363 |
| 2719K | DM3770G_2725 | 0.783067 | 0.976768 | 0.764875 | 4518363 |
| 2753L | BT2849G_1909 | 0.626956 | 0.988297 | 0.619619 | 5329059 |
| 2753L | DM2858G_3735 | 0.520819 | 0.985097 | 0.513057 | 5329059 |
| 991H | 1133Y | 0.888098 | 0.975928 | 0.86672 | 4574307 |
| 991H | 1675L | 0.88192 | 0.982037 | 0.866078 | 4574307 |
| 991H | NR01_83_0.fasta | 0.622196 | 0.930411 | 0.578898 | 4574307 |
| 991H | NR02_1_1.fasta | 0.8166 | 0.917586 | 0.749301 | 4574307 |
| 991H | NR03_uc.fasta | 0.632584 | 0.933684 | 0.590634 | 4574307 |
| 991H | T0609 | 0.86053 | 0.983114 | 0.845999 | 4574307 |
| 991H | T7901 | 0.873763 | 0.923005 | 0.806488 | 4574307 |
| 991H | T7902 | 0.894225 | 0.981671 | 0.877835 | 4574307 |
| 991H | T8402 | 0.880146 | 0.923846 | 0.813119 | 4574307 |
| 991H | T8412 | 0.89513 | 0.979792 | 0.877041 | 4574307 |
| 991H | T8415 | 0.869846 | 0.923357 | 0.803178 | 4574307 |
| 991H | T8513 | 0.885041 | 0.983463 | 0.870405 | 4574307 |
| 991H | T8602 | 0.85937 | 0.922279 | 0.792579 | 4574307 |
| 991H | TBF05_2_0.fasta | 0.792542 | 0.976231 | 0.773704 | 4574307 |
| BS08 | BSG2_2_0.fasta | 0.761704 | 0.992799 | 0.756219 | 4165124 |
| BSC2.fasta | BSG1_1_1.fasta | 0.87084 | 0.965521 | 0.840814 | 4577055 |
| BSC2.fasta | BSG3_2_0.fasta | 0.724032 | 0.958843 | 0.694233 | 4577055 |
| BSC2.fasta | BSG4_1_0.fasta | 0.711961 | 0.970806 | 0.691176 | 4577055 |
| BSG1_1_1.fasta | BSC2.fasta | 0.645775 | 0.973085 | 0.628394 | 6666104 |
| BSG2_2_0.fasta | BS08 | 0.920644 | 0.995137 | 0.916167 | 3513639 |
| BSG2_2_1.fasta | BSC2.fasta | 0.898617 | 0.9748 | 0.875972 | 2557003 |
| BSG2_2_4.fasta | BSC2.fasta | 0.945788 | 0.976468 | 0.923532 | 323727 |
| BSG2_2_9.fasta | BSC2.fasta | 0.904797 | 0.980741 | 0.887372 | 137905 |
| BSG3_2_0.fasta | BSC2.fasta | 0.593708 | 0.973826 | 0.578168 | 6133323 |
| BSG4_1_0.fasta | BSC2.fasta | 0.861395 | 0.974795 | 0.839684 | 3869091 |
| BT2771G_1251 | 2753L | 0.737287 | 0.985666 | 0.726719 | 931086 |
| BT2771G_1266 | 2753L | 0.959437 | 0.99215 | 0.951905 | 1872123 |
| BT2771G_2629 | 2753L | 0.903905 | 0.991576 | 0.896291 | 2667810 |
| BT2849G_1158 | 2753L | 0.784775 | 0.9885 | 0.77575 | 1191456 |
| BT2849G_1418 | 2753L | 0.990501 | 0.992228 | 0.982803 | 16107 |
| BT2849G_1523 | 2753L | 0.540321 | 0.985773 | 0.532634 | 21465 |
| BT2849G_1577 | 2753L | 0.711287 | 0.963563 | 0.68537 | 36732 |
| BT2849G_1909 | 2753L | 0.971633 | 0.991404 | 0.963281 | 3503796 |
| BT2849G_2869 | 2753L | 0.830116 | 0.988927 | 0.820924 | 687471 |
| BT3790G_1208 | 2753L | 0.745048 | 0.982692 | 0.732153 | 402075 |
| BT3790G_1493 | 2753L | 0.768579 | 0.989095 | 0.760198 | 2051745 |
| BT3790G_1981 | 2753L | 0.552787 | 0.959462 | 0.530378 | 64305 |
| BT3790G_2237 | 2753L | 0.933243 | 0.991594 | 0.925398 | 3015375 |
| BT3790G_3135 | 2753L | 0.626175 | 0.981932 | 0.614861 | 732126 |
| DM2722G_1447 | 1133Y | 0.869977 | 0.935091 | 0.813508 | 2119464 |
| DM2722G_1447 | 1675L | 0.863317 | 0.928096 | 0.801241 | 2119464 |
| DM2722G_1447 | 991H | 0.865791 | 0.927151 | 0.802719 | 2119464 |
| DM2722G_1447 | T0609 | 0.859012 | 0.928516 | 0.797606 | 2119464 |
| DM2722G_1447 | T7901 | 0.891632 | 0.983404 | 0.876834 | 2119464 |
| DM2722G_1447 | T7902 | 0.872281 | 0.927808 | 0.809309 | 2119464 |
| DM2722G_1447 | T8402 | 0.891799 | 0.981492 | 0.875294 | 2119464 |
| DM2722G_1447 | T8412 | 0.863001 | 0.926899 | 0.799915 | 2119464 |
| DM2722G_1447 | T8415 | 0.885098 | 0.981571 | 0.868787 | 2119464 |
| DM2722G_1447 | T8513 | 0.864245 | 0.927157 | 0.801291 | 2119464 |
| DM2722G_1447 | T8602 | 0.895983 | 0.985196 | 0.882719 | 2119464 |
| DM2722G_1691 | 2753L | 0.860087 | 0.991096 | 0.852429 | 2544396 |
| DM2722G_1870 | 1133Y | 0.873943 | 0.927325 | 0.810429 | 2047428 |
| DM2722G_1870 | 1675L | 0.87697 | 0.921825 | 0.808413 | 2047428 |
| DM2722G_1870 | 991H | 0.877267 | 0.920066 | 0.807144 | 2047428 |
| DM2722G_1870 | T0609 | 0.878737 | 0.922672 | 0.810786 | 2047428 |
| DM2722G_1870 | T7901 | 0.914987 | 0.981546 | 0.898102 | 2047428 |
| DM2722G_1870 | T7902 | 0.871213 | 0.920104 | 0.801607 | 2047428 |
| DM2722G_1870 | T8402 | 0.900602 | 0.980756 | 0.883271 | 2047428 |
| DM2722G_1870 | T8412 | 0.867036 | 0.919955 | 0.797634 | 2047428 |
| DM2722G_1870 | T8415 | 0.895129 | 0.981002 | 0.878123 | 2047428 |
| DM2722G_1870 | T8513 | 0.861727 | 0.920596 | 0.793302 | 2047428 |
| DM2722G_1870 | T8602 | 0.905362 | 0.982463 | 0.889485 | 2047428 |
| DM2722G_3144 | 2753L | 0.682074 | 0.989466 | 0.674889 | 3191535 |
| DM2722G_497 | 2719K | 0.707764 | 0.988715 | 0.699777 | 515829 |
| DM2722G_579 | 2719K | 0.986046 | 0.99807 | 0.984143 | 4146420 |
| DM2858G_1105 | 1133Y | 0.620173 | 0.946415 | 0.586941 | 1252896 |
| DM2858G_1105 | 1675L | 0.633836 | 0.935645 | 0.593045 | 1252896 |
| DM2858G_1105 | 991H | 0.619454 | 0.931833 | 0.577228 | 1252896 |
| DM2858G_1105 | T0609 | 0.603874 | 0.934476 | 0.564306 | 1252896 |
| DM2858G_1105 | T7901 | 0.64117 | 0.976821 | 0.626308 | 1252896 |
| DM2858G_1105 | T7902 | 0.611744 | 0.933045 | 0.570785 | 1252896 |
| DM2858G_1105 | T8402 | 0.643978 | 0.973645 | 0.627006 | 1252896 |
| DM2858G_1105 | T8412 | 0.605823 | 0.931875 | 0.564551 | 1252896 |
| DM2858G_1105 | T8415 | 0.63788 | 0.974056 | 0.621331 | 1252896 |
| DM2858G_1105 | T8513 | 0.60504 | 0.93013 | 0.562766 | 1252896 |
| DM2858G_1105 | T8602 | 0.654028 | 0.976458 | 0.638631 | 1252896 |
| DM2858G_1458 | 2719K | 0.95337 | 0.996185 | 0.949733 | 4319439 |
| DM2858G_2488 | 1133Y | 0.527697 | 0.915996 | 0.483368 | 31086 |
| DM2858G_2488 | 1675L | 0.509168 | 0.922921 | 0.469922 | 31086 |
| DM2858G_2488 | 991H | 0.519494 | 0.912874 | 0.474233 | 31086 |
| DM2858G_2488 | T7901 | 0.565238 | 0.959991 | 0.542623 | 31086 |
| DM2858G_2488 | T7902 | 0.541884 | 0.911428 | 0.493888 | 31086 |
| DM2858G_2488 | T8402 | 0.539761 | 0.97622 | 0.526925 | 31086 |
| DM2858G_2488 | T8412 | 0.533681 | 0.914949 | 0.488291 | 31086 |
| DM2858G_2488 | T8415 | 0.570546 | 0.97412 | 0.55578 | 31086 |
| DM2858G_2488 | T8602 | 0.569774 | 0.971827 | 0.553722 | 31086 |
| DM2858G_2501 | 2719K | 0.701329 | 0.98137 | 0.688263 | 411372 |
| DM2858G_2907 | 1133Y | 0.630511 | 0.914008 | 0.576292 | 2150778 |
| DM2858G_2907 | 1675L | 0.623165 | 0.90994 | 0.567043 | 2150778 |
| DM2858G_2907 | 991H | 0.622968 | 0.909982 | 0.56689 | 2150778 |
| DM2858G_2907 | T0609 | 0.626758 | 0.910536 | 0.570686 | 2150778 |
| DM2858G_2907 | T7901 | 0.652576 | 0.972146 | 0.634399 | 2150778 |
| DM2858G_2907 | T7902 | 0.624774 | 0.908152 | 0.56739 | 2150778 |
| DM2858G_2907 | T8402 | 0.648725 | 0.972033 | 0.630582 | 2150778 |
| DM2858G_2907 | T8412 | 0.630313 | 0.9102 | 0.573711 | 2150778 |
| DM2858G_2907 | T8415 | 0.646015 | 0.972657 | 0.628351 | 2150778 |
| DM2858G_2907 | T8513 | 0.618733 | 0.909773 | 0.562907 | 2150778 |
| DM2858G_2907 | T8602 | 0.641596 | 0.972786 | 0.624136 | 2150778 |
| DM2858G_3045 | 1133Y | 0.919354 | 0.933049 | 0.857802 | 1967640 |
| DM2858G_3045 | 1675L | 0.914358 | 0.926672 | 0.84731 | 1967640 |
| DM2858G_3045 | 991H | 0.916906 | 0.926561 | 0.849569 | 1967640 |
| DM2858G_3045 | T0609 | 0.916133 | 0.927309 | 0.849538 | 1967640 |
| DM2858G_3045 | T7901 | 0.932526 | 0.984445 | 0.918021 | 1967640 |
| DM2858G_3045 | T7902 | 0.907632 | 0.926104 | 0.840562 | 1967640 |
| DM2858G_3045 | T8402 | 0.928931 | 0.982109 | 0.912311 | 1967640 |
| DM2858G_3045 | T8412 | 0.910497 | 0.925235 | 0.842424 | 1967640 |
| DM2858G_3045 | T8415 | 0.928217 | 0.981995 | 0.911504 | 1967640 |
| DM2858G_3045 | T8513 | 0.908572 | 0.925417 | 0.840808 | 1967640 |
| DM2858G_3045 | T8602 | 0.934802 | 0.986011 | 0.921725 | 1967640 |
| DM2858G_3735 | 2753L | 0.861203 | 0.991039 | 0.853486 | 3409581 |
| DM3770G_1109 | T8415 | 0.528894 | 0.9172 | 0.485102 | 13290 |
| DM3770G_1432 | T7901 | 0.515957 | 0.973758 | 0.502417 | 771279 |
| DM3770G_2006 | 1133Y | 0.936908 | 0.926142 | 0.86771 | 1755333 |
| DM3770G_2006 | 1675L | 0.936995 | 0.924306 | 0.86607 | 1755333 |
| DM3770G_2006 | 991H | 0.942717 | 0.923133 | 0.870253 | 1755333 |
| DM3770G_2006 | T0609 | 0.93762 | 0.923223 | 0.865632 | 1755333 |
| DM3770G_2006 | T7901 | 0.954063 | 0.985646 | 0.940368 | 1755333 |
| DM3770G_2006 | T7902 | 0.937723 | 0.922307 | 0.864868 | 1755333 |
| DM3770G_2006 | T8402 | 0.953564 | 0.983361 | 0.937698 | 1755333 |
| DM3770G_2006 | T8412 | 0.934559 | 0.92214 | 0.861794 | 1755333 |
| DM3770G_2006 | T8415 | 0.956646 | 0.983846 | 0.941192 | 1755333 |
| DM3770G_2006 | T8513 | 0.93782 | 0.922223 | 0.864879 | 1755333 |
| DM3770G_2006 | T8602 | 0.948791 | 0.987314 | 0.936755 | 1755333 |
| DM3770G_2725 | 2719K | 0.915257 | 0.985789 | 0.90225 | 4824342 |
| DM3770G_2751 | 1133Y | 0.892276 | 0.947536 | 0.845464 | 406707 |
| DM3770G_2751 | 1675L | 0.863191 | 0.938519 | 0.810121 | 406707 |
| DM3770G_2751 | 991H | 0.875938 | 0.938369 | 0.821953 | 406707 |
| DM3770G_2751 | T0609 | 0.876034 | 0.938738 | 0.822366 | 406707 |
| DM3770G_2751 | T7901 | 0.893228 | 0.974315 | 0.870285 | 406707 |
| DM3770G_2751 | T7902 | 0.849081 | 0.937778 | 0.796249 | 406707 |
| DM3770G_2751 | T8402 | 0.882879 | 0.971073 | 0.85734 | 406707 |
| DM3770G_2751 | T8412 | 0.869786 | 0.936096 | 0.814203 | 406707 |
| DM3770G_2751 | T8415 | 0.885077 | 0.968797 | 0.85746 | 406707 |
| DM3770G_2751 | T8513 | 0.85316 | 0.936312 | 0.798824 | 406707 |
| DM3770G_2751 | T8602 | 0.901799 | 0.973795 | 0.878167 | 406707 |
| DM3770G_2901 | 2753L | 0.791226 | 0.988497 | 0.782125 | 105042 |
| DM3770G_2983 | 1133Y | 0.741582 | 0.926188 | 0.686844 | 1267746 |
| DM3770G_2983 | 1675L | 0.710092 | 0.918912 | 0.652512 | 1267746 |
| DM3770G_2983 | 991H | 0.734198 | 0.918836 | 0.674608 | 1267746 |
| DM3770G_2983 | T0609 | 0.722201 | 0.918436 | 0.663295 | 1267746 |
| DM3770G_2983 | T7901 | 0.777272 | 0.981042 | 0.762536 | 1267746 |
| DM3770G_2983 | T7902 | 0.721815 | 0.918004 | 0.662629 | 1267746 |
| DM3770G_2983 | T8402 | 0.777461 | 0.980031 | 0.761936 | 1267746 |
| DM3770G_2983 | T8412 | 0.735069 | 0.919615 | 0.67598 | 1267746 |
| DM3770G_2983 | T8415 | 0.74838 | 0.98179 | 0.734752 | 1267746 |
| DM3770G_2983 | T8513 | 0.723786 | 0.918056 | 0.664476 | 1267746 |
| DM3770G_2983 | T8602 | 0.761282 | 0.980425 | 0.74638 | 1267746 |
| DM3770G_3242 | 2753L | 0.770013 | 0.980837 | 0.755257 | 37476 |
| DM3770G_3460 | T8602 | 0.521027 | 0.958985 | 0.499657 | 36453 |
| DM3770G_5 | 2753L | 0.51835 | 0.986173 | 0.511183 | 276537 |
| DM3770G_615 | 1133Y | 0.870698 | 0.943556 | 0.821552 | 1408404 |
| DM3770G_615 | 1675L | 0.879922 | 0.934937 | 0.822672 | 1408404 |
| DM3770G_615 | 991H | 0.878307 | 0.933908 | 0.820258 | 1408404 |
| DM3770G_615 | T0609 | 0.860142 | 0.935645 | 0.804788 | 1408404 |
| DM3770G_615 | T7901 | 0.90391 | 0.980828 | 0.88658 | 1408404 |
| DM3770G_615 | T7902 | 0.872341 | 0.932991 | 0.813886 | 1408404 |
| DM3770G_615 | T8402 | 0.904013 | 0.979725 | 0.885684 | 1408404 |
| DM3770G_615 | T8412 | 0.849266 | 0.932658 | 0.792075 | 1408404 |
| DM3770G_615 | T8415 | 0.908096 | 0.978877 | 0.888914 | 1408404 |
| DM3770G_615 | T8513 | 0.864238 | 0.933212 | 0.806517 | 1408404 |
| DM3770G_615 | T8602 | 0.915869 | 0.982445 | 0.899791 | 1408404 |
| DM3770G_994 | 2719K | 0.553608 | 0.964841 | 0.534144 | 677700 |
| KP2132G_2024 | 2141T | 0.951036 | 0.96525 | 0.917987 | 236499 |
| KP2132G_487 | 2141T | 0.751529 | 0.947831 | 0.712322 | 2396295 |
| KP2132G_543 | 2141T | 0.930654 | 0.947829 | 0.882101 | 7410314 |
| KP2132G_930 | 2141T | 0.913836 | 0.969131 | 0.885627 | 15633 |
| KP2133G_110 | 2141T | 0.970586 | 0.978075 | 0.949306 | 184775 |
| KP2133G_12 | 2141T | 0.852426 | 0.972543 | 0.829021 | 906223 |
| KP2133G_1401 | 2141T | 0.991723 | 0.918486 | 0.910884 | 36851 |
| KP2133G_1537 | 2141T | 0.937146 | 0.974138 | 0.91291 | 1388649 |
| KP2133G_1802 | 2141T | 0.885012 | 0.971645 | 0.859917 | 96236 |
| KP2133G_407 | 2141T | 0.514411 | 0.963889 | 0.495835 | 424581 |
| KP2133G_561 | 2141T | 0.847441 | 0.979638 | 0.830185 | 20805 |
| KP2133G_574 | 2141T | 0.956468 | 0.973746 | 0.931357 | 2478081 |
| KP2133G_581 | 2141T | 0.968286 | 0.976388 | 0.945423 | 52311 |
| KP2133G_742 | 2141T | 0.846229 | 0.972483 | 0.822943 | 860714 |
| KP3700G_1264 | 2141T | 0.916918 | 0.979339 | 0.897974 | 5185779 |
| KP3700G_1558 | 2141T | 0.715557 | 0.973669 | 0.696716 | 1902657 |
| KP3700G_2285 | 2141T | 0.523456 | 0.953913 | 0.499331 | 94260 |
| KP3700G_254 | 2141T | 0.65172 | 0.96095 | 0.62627 | 67506 |
| NR01_83_0.fasta | 1133Y | 0.67502 | 0.946155 | 0.638674 | 6971531 |
| NR01_83_0.fasta | 1675L | 0.6816 | 0.945046 | 0.644143 | 6971531 |
| NR01_83_0.fasta | 991H | 0.694159 | 0.946682 | 0.657148 | 6971531 |
| NR01_83_0.fasta | T0609 | 0.660568 | 0.946208 | 0.625035 | 6971531 |
| NR01_83_0.fasta | T7901 | 0.689513 | 0.945465 | 0.65191 | 6971531 |
| NR01_83_0.fasta | T7902 | 0.686353 | 0.946113 | 0.649367 | 6971531 |
| NR01_83_0.fasta | T8402 | 0.687244 | 0.944579 | 0.649156 | 6971531 |
| NR01_83_0.fasta | T8412 | 0.693179 | 0.945101 | 0.655124 | 6971531 |
| NR01_83_0.fasta | T8415 | 0.679432 | 0.944509 | 0.64173 | 6971531 |
| NR01_83_0.fasta | T8513 | 0.713256 | 0.953623 | 0.680177 | 6971531 |
| NR01_83_0.fasta | T8602 | 0.674679 | 0.944171 | 0.637012 | 6971531 |
| NR01_uc.fasta | 1133Y | 0.89634 | 0.937844 | 0.840627 | 1878871 |
| NR01_uc.fasta | 1675L | 0.900039 | 0.932247 | 0.839059 | 1878871 |
| NR01_uc.fasta | 991H | 0.896425 | 0.932008 | 0.835475 | 1878871 |
| NR01_uc.fasta | T0609 | 0.894596 | 0.932416 | 0.834136 | 1878871 |
| NR01_uc.fasta | T7901 | 0.908853 | 0.979432 | 0.89016 | 1878871 |
| NR01_uc.fasta | T7902 | 0.898114 | 0.931294 | 0.836408 | 1878871 |
| NR01_uc.fasta | T8402 | 0.912015 | 0.97896 | 0.892826 | 1878871 |
| NR01_uc.fasta | T8412 | 0.891515 | 0.931243 | 0.830217 | 1878871 |
| NR01_uc.fasta | T8415 | 0.912593 | 0.97762 | 0.892169 | 1878871 |
| NR01_uc.fasta | T8513 | 0.894387 | 0.932666 | 0.834164 | 1878871 |
| NR01_uc.fasta | T8602 | 0.915191 | 0.978577 | 0.895585 | 1878871 |
| NR02_1_1.fasta | 1133Y | 0.668416 | 0.931331 | 0.622517 | 6001350 |
| NR02_1_1.fasta | 1675L | 0.679296 | 0.925157 | 0.628455 | 6001350 |
| NR02_1_1.fasta | 991H | 0.683963 | 0.924842 | 0.632558 | 6001350 |
| NR02_1_1.fasta | T0609 | 0.658962 | 0.925538 | 0.609894 | 6001350 |
| NR02_1_1.fasta | T7901 | 0.692028 | 0.983431 | 0.680562 | 6001350 |
| NR02_1_1.fasta | T7902 | 0.674652 | 0.92368 | 0.623163 | 6001350 |
| NR02_1_1.fasta | T8402 | 0.69478 | 0.979676 | 0.680659 | 6001350 |
| NR02_1_1.fasta | T8412 | 0.675651 | 0.923138 | 0.623719 | 6001350 |
| NR02_1_1.fasta | T8415 | 0.69717 | 0.980238 | 0.683393 | 6001350 |
| NR02_1_1.fasta | T8513 | 0.682099 | 0.924662 | 0.630711 | 6001350 |
| NR02_1_1.fasta | T8602 | 0.67917 | 0.981851 | 0.666844 | 6001350 |
| NR03_1_5.fasta | 1133Y | 0.876318 | 0.936051 | 0.820278 | 62313 |
| NR03_1_5.fasta | 1675L | 0.910404 | 0.960303 | 0.874264 | 62313 |
| NR03_1_5.fasta | 991H | 0.932117 | 0.959799 | 0.894645 | 62313 |
| NR03_1_5.fasta | T0609 | 0.818304 | 0.954325 | 0.780928 | 62313 |
| NR03_1_5.fasta | T7901 | 0.955996 | 0.947273 | 0.905589 | 62313 |
| NR03_1_5.fasta | T7902 | 0.897549 | 0.955068 | 0.85722 | 62313 |
| NR03_1_5.fasta | T8402 | 0.880506 | 0.973973 | 0.857589 | 62313 |
| NR03_1_5.fasta | T8412 | 0.876318 | 0.940355 | 0.82405 | 62313 |
| NR03_1_5.fasta | T8415 | 0.880506 | 0.973992 | 0.857606 | 62313 |
| NR03_1_5.fasta | T8513 | 0.824804 | 0.947214 | 0.781266 | 62313 |
| NR03_1_5.fasta | T8602 | 0.862067 | 0.958841 | 0.826585 | 62313 |
| NR03_3_0.fasta | 1133Y | 0.668093 | 0.935554 | 0.625037 | 1713843 |
| NR03_3_0.fasta | 1675L | 0.681904 | 0.932979 | 0.636202 | 1713843 |
| NR03_3_0.fasta | 991H | 0.673016 | 0.932529 | 0.627607 | 1713843 |
| NR03_3_0.fasta | T0609 | 0.665706 | 0.93191 | 0.620378 | 1713843 |
| NR03_3_0.fasta | T7901 | 0.675478 | 0.965013 | 0.651845 | 1713843 |
| NR03_3_0.fasta | T7902 | 0.673639 | 0.932215 | 0.627976 | 1713843 |
| NR03_3_0.fasta | T8402 | 0.666042 | 0.962142 | 0.640827 | 1713843 |
| NR03_3_0.fasta | T8412 | 0.666094 | 0.931251 | 0.620301 | 1713843 |
| NR03_3_0.fasta | T8415 | 0.669292 | 0.963475 | 0.644846 | 1713843 |
| NR03_3_0.fasta | T8513 | 0.684772 | 0.933819 | 0.639453 | 1713843 |
| NR03_3_0.fasta | T8602 | 0.661184 | 0.963555 | 0.637087 | 1713843 |
| NR03_uc.fasta | 1133Y | 0.613876 | 0.946813 | 0.581226 | 7380431 |
| NR03_uc.fasta | 1675L | 0.620773 | 0.945993 | 0.587247 | 7380431 |
| NR03_uc.fasta | 991H | 0.638711 | 0.945861 | 0.604132 | 7380431 |
| NR03_uc.fasta | T0609 | 0.607121 | 0.945044 | 0.573756 | 7380431 |
| NR03_uc.fasta | T7901 | 0.622761 | 0.95804 | 0.59663 | 7380431 |
| NR03_uc.fasta | T7902 | 0.628333 | 0.945183 | 0.59389 | 7380431 |
| NR03_uc.fasta | T8402 | 0.638135 | 0.957549 | 0.611046 | 7380431 |
| NR03_uc.fasta | T8412 | 0.636491 | 0.943442 | 0.600492 | 7380431 |
| NR03_uc.fasta | T8415 | 0.628302 | 0.956369 | 0.600889 | 7380431 |
| NR03_uc.fasta | T8513 | 0.642384 | 0.946389 | 0.607945 | 7380431 |
| NR03_uc.fasta | T8602 | 0.615265 | 0.956948 | 0.588777 | 7380431 |
| T0609 | 1133Y | 0.885222 | 0.974391 | 0.862552 | 4392680 |
| T0609 | 1675L | 0.90846 | 0.978659 | 0.889073 | 4392680 |
| T0609 | 991H | 0.896712 | 0.982882 | 0.881362 | 4392680 |
| T0609 | NR01_83_0.fasta | 0.627597 | 0.93272 | 0.585372 | 4392680 |
| T0609 | NR02_1_1.fasta | 0.827633 | 0.920637 | 0.76195 | 4392680 |
| T0609 | NR03_uc.fasta | 0.636922 | 0.935509 | 0.595846 | 4392680 |
| T0609 | T7901 | 0.873915 | 0.924175 | 0.80765 | 4392680 |
| T0609 | T7902 | 0.894391 | 0.981676 | 0.878002 | 4392680 |
| T0609 | T8402 | 0.876731 | 0.926249 | 0.812071 | 4392680 |
| T0609 | T8412 | 0.895122 | 0.97822 | 0.875626 | 4392680 |
| T0609 | T8415 | 0.884963 | 0.923698 | 0.817439 | 4392680 |
| T0609 | T8513 | 0.892209 | 0.982359 | 0.87647 | 4392680 |
| T0609 | T8602 | 0.870858 | 0.923299 | 0.804062 | 4392680 |
| T0609 | TBF05_2_0.fasta | 0.809401 | 0.97434 | 0.788632 | 4392680 |
| T7901 | 1133Y | 0.889122 | 0.930659 | 0.827469 | 4485681 |
| T7901 | 1675L | 0.874732 | 0.924164 | 0.808396 | 4485681 |
| T7901 | 991H | 0.891699 | 0.923504 | 0.823488 | 4485681 |
| T7901 | NR01_83_0.fasta | 0.631319 | 0.946123 | 0.597305 | 4485681 |
| T7901 | NR02_1_1.fasta | 0.840667 | 0.979563 | 0.823486 | 4485681 |
| T7901 | NR03_uc.fasta | 0.626208 | 0.95685 | 0.599187 | 4485681 |
| T7901 | T0609 | 0.857654 | 0.924544 | 0.792939 | 4485681 |
| T7901 | T7902 | 0.8819 | 0.922027 | 0.813136 | 4485681 |
| T7901 | T8402 | 0.910335 | 0.981911 | 0.893868 | 4485681 |
| T7901 | T8412 | 0.880605 | 0.921929 | 0.811855 | 4485681 |
| T7901 | T8415 | 0.902292 | 0.980763 | 0.884935 | 4485681 |
| T7901 | T8513 | 0.87447 | 0.92361 | 0.807669 | 4485681 |
| T7901 | T8602 | 0.901004 | 0.982316 | 0.885071 | 4485681 |
| T7901 | TBF05_2_0.fasta | 0.796251 | 0.918111 | 0.731047 | 4485681 |
| T7902 | 1133Y | 0.846907 | 0.976243 | 0.826787 | 4679711 |
| T7902 | 1675L | 0.902333 | 0.985913 | 0.889622 | 4679711 |
| T7902 | 991H | 0.879235 | 0.982723 | 0.864044 | 4679711 |
| T7902 | NR01_83_0.fasta | 0.616936 | 0.927803 | 0.572395 | 4679711 |
| T7902 | NR02_1_1.fasta | 0.804973 | 0.917494 | 0.738558 | 4679711 |
| T7902 | NR03_uc.fasta | 0.625576 | 0.933852 | 0.584195 | 4679711 |
| T7902 | T0609 | 0.853667 | 0.979927 | 0.836531 | 4679711 |
| T7902 | T7901 | 0.853014 | 0.921915 | 0.786406 | 4679711 |
| T7902 | T8402 | 0.881667 | 0.921382 | 0.812352 | 4679711 |
| T7902 | T8412 | 0.887045 | 0.979357 | 0.868734 | 4679711 |
| T7902 | T8415 | 0.875878 | 0.921236 | 0.80689 | 4679711 |
| T7902 | T8513 | 0.882228 | 0.981673 | 0.866059 | 4679711 |
| T7902 | T8602 | 0.839603 | 0.922374 | 0.774428 | 4679711 |
| T7902 | TBF05_2_0.fasta | 0.770507 | 0.976281 | 0.752231 | 4679711 |
| T8402 | 1133Y | 0.880803 | 0.931718 | 0.82066 | 4501956 |
| T8402 | 1675L | 0.871153 | 0.925977 | 0.806668 | 4501956 |
| T8402 | 991H | 0.889551 | 0.925788 | 0.823536 | 4501956 |
| T8402 | NR01_83_0.fasta | 0.624453 | 0.944341 | 0.589697 | 4501956 |
| T8402 | NR02_1_1.fasta | 0.838445 | 0.976034 | 0.818351 | 4501956 |
| T8402 | NR03_uc.fasta | 0.640692 | 0.955863 | 0.612414 | 4501956 |
| T8402 | T0609 | 0.855565 | 0.926143 | 0.792376 | 4501956 |
| T8402 | T7901 | 0.911137 | 0.981586 | 0.894359 | 4501956 |
| T8402 | T7902 | 0.891047 | 0.9231 | 0.822525 | 4501956 |
| T8402 | T8412 | 0.891846 | 0.923396 | 0.823527 | 4501956 |
| T8402 | T8415 | 0.912321 | 0.979713 | 0.893813 | 4501956 |
| T8402 | T8513 | 0.879714 | 0.925564 | 0.814232 | 4501956 |
| T8402 | T8602 | 0.898003 | 0.980678 | 0.880652 | 4501956 |
| T8402 | TBF05_2_0.fasta | 0.788936 | 0.918738 | 0.724825 | 4501956 |
| T8412 | 1133Y | 0.890765 | 0.972143 | 0.865951 | 4467551 |
| T8412 | 1675L | 0.889963 | 0.979045 | 0.871314 | 4467551 |
| T8412 | 991H | 0.920565 | 0.98011 | 0.902255 | 4467551 |
| T8412 | NR01_83_0.fasta | 0.635071 | 0.930336 | 0.590829 | 4467551 |
| T8412 | NR02_1_1.fasta | 0.824026 | 0.916729 | 0.755409 | 4467551 |
| T8412 | NR03_uc.fasta | 0.638637 | 0.933147 | 0.595942 | 4467551 |
| T8412 | T0609 | 0.882977 | 0.978528 | 0.864018 | 4467551 |
| T8412 | T7901 | 0.891578 | 0.921872 | 0.821921 | 4467551 |
| T8412 | T7902 | 0.917881 | 0.979589 | 0.899146 | 4467551 |
| T8412 | T8402 | 0.901942 | 0.923388 | 0.832842 | 4467551 |
| T8412 | T8415 | 0.883512 | 0.921931 | 0.814537 | 4467551 |
| T8412 | T8513 | 0.918563 | 0.979996 | 0.900188 | 4467551 |
| T8412 | T8602 | 0.8739 | 0.922244 | 0.805949 | 4467551 |
| T8412 | TBF05_2_0.fasta | 0.816165 | 0.974634 | 0.795462 | 4467551 |
| T8415 | 1133Y | 0.87938 | 0.93364 | 0.821024 | 4476275 |
| T8415 | 1675L | 0.898434 | 0.926844 | 0.832708 | 4476275 |
| T8415 | 991H | 0.884153 | 0.925735 | 0.818491 | 4476275 |
| T8415 | NR01_83_0.fasta | 0.636397 | 0.946789 | 0.602534 | 4476275 |
| T8415 | NR02_1_1.fasta | 0.852261 | 0.977522 | 0.833104 | 4476275 |
| T8415 | NR03_uc.fasta | 0.641977 | 0.956194 | 0.613855 | 4476275 |
| T8415 | T0609 | 0.86685 | 0.924166 | 0.801113 | 4476275 |
| T8415 | T7901 | 0.906795 | 0.981376 | 0.889907 | 4476275 |
| T8415 | T7902 | 0.884674 | 0.924949 | 0.818278 | 4476275 |
| T8415 | T8402 | 0.91731 | 0.981868 | 0.900677 | 4476275 |
| T8415 | T8412 | 0.877572 | 0.924005 | 0.810881 | 4476275 |
| T8415 | T8513 | 0.876244 | 0.925177 | 0.810681 | 4476275 |
| T8415 | T8602 | 0.889734 | 0.982798 | 0.874429 | 4476275 |
| T8415 | TBF05_2_0.fasta | 0.779922 | 0.91913 | 0.71685 | 4476275 |
| T8513 | 1133Y | 0.882059 | 0.974339 | 0.859424 | 4559547 |
| T8513 | 1675L | 0.889501 | 0.981425 | 0.872979 | 4559547 |
| T8513 | 991H | 0.894236 | 0.983704 | 0.879664 | 4559547 |
| T8513 | NR01_83_0.fasta | 0.649385 | 0.938047 | 0.609154 | 4559547 |
| T8513 | NR02_1_1.fasta | 0.817981 | 0.917444 | 0.750452 | 4559547 |
| T8513 | NR03_uc.fasta | 0.635693 | 0.936753 | 0.595487 | 4559547 |
| T8513 | T0609 | 0.861942 | 0.982137 | 0.846545 | 4559547 |
| T8513 | T7901 | 0.870331 | 0.92317 | 0.803463 | 4559547 |
| T8513 | T7902 | 0.895824 | 0.981855 | 0.879569 | 4559547 |
| T8513 | T8402 | 0.873841 | 0.925228 | 0.808502 | 4559547 |
| T8513 | T8412 | 0.901188 | 0.980463 | 0.883581 | 4559547 |
| T8513 | T8415 | 0.863506 | 0.924296 | 0.798135 | 4559547 |
| T8513 | T8602 | 0.862103 | 0.920981 | 0.79398 | 4559547 |
| T8513 | TBF05_2_0.fasta | 0.798762 | 0.975963 | 0.779562 | 4559547 |
| T8602 | 1133Y | 0.892922 | 0.931257 | 0.83154 | 4444880 |
| T8602 | 1675L | 0.866881 | 0.924539 | 0.801465 | 4444880 |
| T8602 | 991H | 0.885145 | 0.922655 | 0.816683 | 4444880 |
| T8602 | NR01_83_0.fasta | 0.62017 | 0.945379 | 0.586296 | 4444880 |
| T8602 | NR02_1_1.fasta | 0.835341 | 0.977696 | 0.81671 | 4444880 |
| T8602 | NR03_uc.fasta | 0.626023 | 0.955289 | 0.598033 | 4444880 |
| T8602 | T0609 | 0.862304 | 0.923203 | 0.796082 | 4444880 |
| T8602 | T7901 | 0.909376 | 0.982195 | 0.893185 | 4444880 |
| T8602 | T7902 | 0.87942 | 0.922876 | 0.811596 | 4444880 |
| T8602 | T8402 | 0.910518 | 0.9807 | 0.892945 | 4444880 |
| T8602 | T8412 | 0.877947 | 0.922503 | 0.809909 | 4444880 |
| T8602 | T8415 | 0.896389 | 0.981936 | 0.880197 | 4444880 |
| T8602 | T8513 | 0.881462 | 0.921479 | 0.812249 | 4444880 |
| T8602 | TBF05_2_0.fasta | 0.80329 | 0.917094 | 0.736692 | 4444880 |
| TBF02_1_1.fasta | 1162T | 0.960455 | 0.984263 | 0.94534 | 450549 |
| TBF02_3_0.fasta | 1133Y | 0.8044 | 0.930773 | 0.748714 | 3234669 |
| TBF02_3_0.fasta | 1675L | 0.788952 | 0.924999 | 0.72978 | 3234669 |
| TBF02_3_0.fasta | 991H | 0.808884 | 0.924883 | 0.748123 | 3234669 |
| TBF02_3_0.fasta | T0609 | 0.783988 | 0.925451 | 0.725542 | 3234669 |
| TBF02_3_0.fasta | T7901 | 0.820008 | 0.976303 | 0.800576 | 3234669 |
| TBF02_3_0.fasta | T7902 | 0.794959 | 0.924469 | 0.734915 | 3234669 |
| TBF02_3_0.fasta | T8402 | 0.813269 | 0.975412 | 0.793272 | 3234669 |
| TBF02_3_0.fasta | T8412 | 0.802364 | 0.923339 | 0.740854 | 3234669 |
| TBF02_3_0.fasta | T8415 | 0.798753 | 0.975965 | 0.779555 | 3234669 |
| TBF02_3_0.fasta | T8513 | 0.798417 | 0.922979 | 0.736922 | 3234669 |
| TBF02_3_0.fasta | T8602 | 0.813756 | 0.978318 | 0.796112 | 3234669 |
| TBF03_2_0.fasta | 1133Y | 0.77877 | 0.972509 | 0.757361 | 4012364 |
| TBF03_2_0.fasta | 1675L | 0.769773 | 0.97976 | 0.754193 | 4012364 |
| TBF03_2_0.fasta | 991H | 0.792989 | 0.980324 | 0.777386 | 4012364 |
| TBF03_2_0.fasta | T0609 | 0.75793 | 0.979196 | 0.742162 | 4012364 |
| TBF03_2_0.fasta | T7901 | 0.77334 | 0.919818 | 0.711332 | 4012364 |
| TBF03_2_0.fasta | T7902 | 0.784174 | 0.980219 | 0.768662 | 4012364 |
| TBF03_2_0.fasta | T8402 | 0.768395 | 0.921958 | 0.708428 | 4012364 |
| TBF03_2_0.fasta | T8412 | 0.785248 | 0.97951 | 0.769158 | 4012364 |
| TBF03_2_0.fasta | T8415 | 0.752277 | 0.921598 | 0.693297 | 4012364 |
| TBF03_2_0.fasta | T8513 | 0.781884 | 0.981158 | 0.767152 | 4012364 |
| TBF03_2_0.fasta | T8602 | 0.75842 | 0.919919 | 0.697685 | 4012364 |
| TBF05_2_0.fasta | 1133Y | 0.92992 | 0.973602 | 0.905372 | 4056823 |
| TBF05_2_0.fasta | 1675L | 0.91262 | 0.980719 | 0.895024 | 4056823 |
| TBF05_2_0.fasta | 991H | 0.935601 | 0.981484 | 0.918277 | 4056823 |
| TBF05_2_0.fasta | T0609 | 0.915587 | 0.980034 | 0.897306 | 4056823 |
| TBF05_2_0.fasta | T7901 | 0.920769 | 0.92287 | 0.84975 | 4056823 |
| TBF05_2_0.fasta | T7902 | 0.923265 | 0.981124 | 0.905837 | 4056823 |
| TBF05_2_0.fasta | T8402 | 0.912109 | 0.923725 | 0.842538 | 4056823 |
| TBF05_2_0.fasta | T8412 | 0.933926 | 0.98047 | 0.915686 | 4056823 |
| TBF05_2_0.fasta | T8415 | 0.90229 | 0.923779 | 0.833517 | 4056823 |
| TBF05_2_0.fasta | T8513 | 0.932749 | 0.982141 | 0.916091 | 4056823 |
| TBF05_2_0.fasta | T8602 | 0.918734 | 0.921887 | 0.846969 | 4056823 |
| TBF05_uc.fasta | 1133Y | 0.659554 | 0.965507 | 0.636804 | 198111 |
| TBF05_uc.fasta | 1675L | 0.646047 | 0.977428 | 0.631464 | 198111 |
| TBF05_uc.fasta | 991H | 0.640762 | 0.978234 | 0.626815 | 198111 |
| TBF05_uc.fasta | T0609 | 0.667929 | 0.972741 | 0.649722 | 198111 |
| TBF05_uc.fasta | T7901 | 0.634311 | 0.91599 | 0.581023 | 198111 |
| TBF05_uc.fasta | T7902 | 0.630964 | 0.977544 | 0.616795 | 198111 |
| TBF05_uc.fasta | T8402 | 0.668655 | 0.919369 | 0.614741 | 198111 |
| TBF05_uc.fasta | T8412 | 0.665173 | 0.966466 | 0.642867 | 198111 |
| TBF05_uc.fasta | T8415 | 0.635492 | 0.916043 | 0.582138 | 198111 |
| TBF05_uc.fasta | T8513 | 0.631192 | 0.97748 | 0.616978 | 198111 |
| TBF05_uc.fasta | T8602 | 0.644336 | 0.912996 | 0.588276 | 198111 |
| TBF07_1_1.fasta | 1162T | 0.895614 | 0.984754 | 0.881959 | 743952 |
| TBF09_17_0.fasta | 1133Y | 0.953758 | 0.971264 | 0.926351 | 1861029 |
| TBF09_17_0.fasta | 1675L | 0.939717 | 0.978883 | 0.919873 | 1861029 |
| TBF09_17_0.fasta | 991H | 0.966973 | 0.979451 | 0.947103 | 1861029 |
| TBF09_17_0.fasta | T0609 | 0.930403 | 0.97855 | 0.910446 | 1861029 |
| TBF09_17_0.fasta | T7901 | 0.943027 | 0.922276 | 0.869731 | 1861029 |
| TBF09_17_0.fasta | T7902 | 0.956445 | 0.979964 | 0.937282 | 1861029 |
| TBF09_17_0.fasta | T8402 | 0.937665 | 0.924468 | 0.866841 | 1861029 |
| TBF09_17_0.fasta | T8412 | 0.96116 | 0.979005 | 0.94098 | 1861029 |
| TBF09_17_0.fasta | T8415 | 0.920284 | 0.923824 | 0.85018 | 1861029 |
| TBF09_17_0.fasta | T8513 | 0.955093 | 0.979827 | 0.935826 | 1861029 |
| TBF09_17_0.fasta | T8602 | 0.934865 | 0.921081 | 0.861086 | 1861029 |
| TBF09_2_0.fasta | 1162T | 0.943394 | 0.981959 | 0.926374 | 294653 |
