# Supplementary material for: Secondary Metabolism in the Gill Microbiota of Shipworms (Teredinidae) as Revealed by Comparison of Metagenomes and Nearly Complete Symbiont Genomes
Source: mSystems. 2020 Jun 30;5(3):e00261-20. doi: 10.1128/mSystems.00261-20 (PMC7329324; doi:10.1128/mSystems.00261-20)
Supplement: TABLE S3 [file mSystems.00261-20-st003.docx]

| Query sequence | Subject sequence | %Ident | length | evalue | qcovs | qlen | slen |
| --- | --- | --- | --- | --- | --- | --- | --- |
| NODE_14347_length_5660_cov_6.146055 | NODE_31934_length_3870_cov_17.413977 | 94.28 | 3883 | 0 | 68 | 5660 | 3870 |
| NODE_23307_length_4536_cov_6.299660 | NODE_23527_length_4515_cov_3.470414 | 94.37 | 3377 | 0 | 83 | 4536 | 4515 |
| NODE_23527_length_4515_cov_3.470414 | NODE_23307_length_4536_cov_6.299660 | 94.37 | 3377 | 0 | 83 | 4515 | 4536 |
| NODE_25141_length_4367_cov_9.751766 | NODE_53552_length_2897_cov_8.209654 | 95.48 | 2897 | 0 | 66 | 4367 | 2897 |
| NODE_31934_length_3870_cov_17.413977 | NODE_14347_length_5660_cov_6.146055 | 94.28 | 3883 | 0 | 100 | 3870 | 5660 |
| NODE_35626_length_3655_cov_13.375212 | NODE_36097_length_3631_cov_8.919088 | 94.18 | 3661 | 0 | 100 | 3655 | 3631 |
| NODE_36097_length_3631_cov_8.919088 | NODE_35626_length_3655_cov_13.375212 | 94.18 | 3661 | 0 | 100 | 3631 | 3655 |
| NODE_38563_length_3503_cov_8.494973 | NODE_39659_length_3448_cov_13.046889 | 92.9 | 3465 | 0 | 98 | 3503 | 3448 |
| NODE_39659_length_3448_cov_13.046889 | NODE_38563_length_3503_cov_8.494973 | 92.9 | 3464 | 0 | 100 | 3448 | 3503 |
| NODE_40202_length_3424_cov_11.970027 | NODE_67412_length_2505_cov_5.815856 | 96.77 | 2507 | 0 | 73 | 3424 | 2505 |
| NODE_41049_length_3384_cov_11.921851 | NODE_66289_length_2532_cov_14.121526 | 97.67 | 2532 | 0 | 75 | 3384 | 2532 |
| NODE_51053_length_2982_cov_12.870675 | NODE_73070_length_2374_cov_7.126054 | 96.46 | 2374 | 0 | 80 | 2982 | 2374 |
| NODE_51562_length_2964_cov_4.371790 | NODE_62798_length_2623_cov_3.187450 | 95.89 | 2629 | 0 | 88 | 2964 | 2623 |
| NODE_53552_length_2897_cov_8.209654 | NODE_25141_length_4367_cov_9.751766 | 95.48 | 2897 | 0 | 100 | 2897 | 4367 |
| NODE_56563_length_2800_cov_13.415454 | NODE_67976_length_2491_cov_5.948101 | 95.54 | 2488 | 0 | 89 | 2800 | 2491 |
| NODE_60745_length_2677_cov_6.553208 | NODE_61584_length_2655_cov_4.045383 | 96.95 | 1806 | 0 | 67 | 2677 | 2655 |
| NODE_61584_length_2655_cov_4.045383 | NODE_60745_length_2677_cov_6.553208 | 96.95 | 1806 | 0 | 68 | 2655 | 2677 |
| NODE_62798_length_2623_cov_3.187450 | NODE_51562_length_2964_cov_4.371790 | 95.89 | 2629 | 0 | 99 | 2623 | 2964 |
| NODE_62828_length_2621_cov_13.203200 | NODE_68217_length_2485_cov_15.815990 | 94 | 2485 | 0 | 95 | 2621 | 2485 |
| NODE_64237_length_2585_cov_4.464692 | NODE_79312_length_2246_cov_3.968471 | 93.8 | 1840 | 0 | 71 | 2585 | 2246 |
| NODE_66289_length_2532_cov_14.121526 | NODE_41049_length_3384_cov_11.921851 | 97.67 | 2532 | 0 | 100 | 2532 | 3384 |
| NODE_66682_length_2523_cov_4.220649 | NODE_70658_length_2428_cov_4.239272 | 95.65 | 2025 | 0 | 80 | 2523 | 2428 |
| NODE_67412_length_2505_cov_5.815856 | NODE_40202_length_3424_cov_11.970027 | 96.77 | 2507 | 0 | 100 | 2505 | 3424 |
| NODE_67878_length_2494_cov_4.392752 | NODE_69000_length_2466_cov_4.622601 | 96.47 | 1614 | 0 | 65 | 2494 | 2466 |
| NODE_67976_length_2491_cov_5.948101 | NODE_56563_length_2800_cov_13.415454 | 95.54 | 2488 | 0 | 99 | 2491 | 2800 |
| NODE_68217_length_2485_cov_15.815990 | NODE_62828_length_2621_cov_13.203200 | 94 | 2485 | 0 | 100 | 2485 | 2621 |
| NODE_69000_length_2466_cov_4.622601 | NODE_67878_length_2494_cov_4.392752 | 96.47 | 1614 | 0 | 65 | 2466 | 2494 |
| NODE_69500_length_2454_cov_10.399914 | NODE_69501_length_2454_cov_9.504929 | 97.6 | 2454 | 0 | 100 | 2454 | 2454 |
| NODE_69501_length_2454_cov_9.504929 | NODE_69500_length_2454_cov_10.399914 | 97.6 | 2454 | 0 | 100 | 2454 | 2454 |
| NODE_70658_length_2428_cov_4.239272 | NODE_66682_length_2523_cov_4.220649 | 95.65 | 2025 | 0 | 83 | 2428 | 2523 |
| NODE_72738_length_2381_cov_15.003540 | NODE_73067_length_2374_cov_13.619174 | 97.31 | 2382 | 0 | 100 | 2381 | 2374 |
| NODE_73028_length_2376_cov_2.580488 | NODE_74717_length_2339_cov_8.961226 | 94.38 | 2009 | 0 | 84 | 2376 | 2339 |
| NODE_73067_length_2374_cov_13.619174 | NODE_72738_length_2381_cov_15.003540 | 97.31 | 2382 | 0 | 100 | 2374 | 2381 |
| NODE_73070_length_2374_cov_7.126054 | NODE_51053_length_2982_cov_12.870675 | 96.46 | 2374 | 0 | 100 | 2374 | 2982 |
| NODE_74717_length_2339_cov_8.961226 | NODE_73028_length_2376_cov_2.580488 | 94.38 | 2009 | 0 | 85 | 2339 | 2376 |
| NODE_79312_length_2246_cov_3.968471 | NODE_64237_length_2585_cov_4.464692 | 93.8 | 1840 | 0 | 81 | 2246 | 2585 |
| NODE_81302_length_2208_cov_11.453282 | NODE_86577_length_2114_cov_8.161565 | 95.36 | 2114 | 0 | 96 | 2208 | 2114 |
| NODE_81429_length_2206_cov_8.507434 | NODE_83204_length_2173_cov_5.881579 | 93.25 | 2207 | 0 | 100 | 2206 | 2173 |
| NODE_83204_length_2173_cov_5.881579 | NODE_81429_length_2206_cov_8.507434 | 93.25 | 2207 | 0 | 100 | 2173 | 2206 |
| NODE_86577_length_2114_cov_8.161565 | NODE_81302_length_2208_cov_11.453282 | 95.36 | 2114 | 0 | 100 | 2114 | 2208 |
