# Supplementary material for: Secondary Metabolism in the Gill Microbiota of Shipworms (Teredinidae) as Revealed by Comparison of Metagenomes and Nearly Complete Symbiont Genomes
Source: mSystems. 2020 Jun 30;5(3):e00261-20. doi: 10.1128/mSystems.00261-20 (PMC7329324; doi:10.1128/mSystems.00261-20)
Supplement: TABLE S4 [file mSystems.00261-20-st004.docx]

| **GCF_1** | **cf_fatty_acid-t1pks-nrps** | **GCF_62** | **terpene** |
| --- | --- | --- | --- |
| **GCF_2** | bacteriocin-transatpks-t1pks-nrps | GCF_63 | terpene |
| **GCF_3** | cf_fatty_acid-transatpks-t1pks-nrps | GCF_64 | terpene |
| **GCF_4** | t1pks-cf_saccharide-nrps | GCF_65 | terpene |
| **GCF_5** | terpene-arylpolyene | GCF_66 | t1pks |
| **GCF_6** | transatpks-cf_saccharide-nrps | GCF_67 | t1pks |
| **GCF_7** | nrps | GCF_68 | t1pks |
| **GCF_8** | cf_fatty_acid-nrps_(tunerbactin) | GCF_69 | t1pks |
| **GCF_9** | t1pks | GCF_70 | t1pks-PUFA |
| **GCF_10** | hserlactone-transatpks-nrps | GCF_71 | t1pks-nrps |
| **GCF_11** | transatpks_(tartrolon) | GCF_72 | t1pks-nrps |
| **GCF_12** | transatpks-nrps | GCF_73 | t1pks-nrps |
| **GCF_13** | t1pks-nrps | GCF_74 | t1pks-nrps |
| **GCF_14** | siderophore | GCF_75 | t1pks-nrps |
| **GCF_15** | transatpks-otherks | GCF_76 | t1pks-cf_saccharide-nrps |
| **GCF_16** | t1pks-nrps | GCF_77 | t1pks-cf_saccharide-nrps |
| **GCF_17** | nrps | GCF_78 | t1pks-cf_fatty_acid |
| **GCF_18** | transatpks | GCF_79 | siderophore |
| **GCF_19** | t1pks | GCF_80 | nrps-transatpks-otherks |
| **GCF_20** | nrps | GCF_81 | nrps |
| **GCF_21** | transatpks | GCF_82 | nrps |
| **GCF_22** | t1pks | GCF_83 | nrps |
| **GCF_23** | siderophore | GCF_84 | nrps |
| **GCF_24** | nrps | GCF_85 | nrps |
| **GCF_25** | nrps | GCF_86 | nrps |
| **GCF_26** | transatpks | GCF_87 | nrps |
| **GCF_27** | transatpks-otherks-nrps | GCF_88 | nrps |
| **GCF_28** | nrps | GCF_89 | nrps |
| **GCF_29** | nrps | GCF_90 | nrps |
| **GCF_30** | nrps | GCF_91 | nrps |
| **GCF_31** | nrps | GCF_92 | nrps |
| **GCF_32** | transatpks | GCF_93 | nrps |
| **GCF_33** | transatpks-t1pks-nrps | GCF_94 | nrps |
| **GCF_34** | thiopeptide-hserlactone | GCF_95 | nrps |
| **GCF_35** | t1pks-cf_saccharide-nrps | GCF_96 | nrps |
| **GCF_36** | t1pks-nrps | GCF_97 | nrps |
| **GCF_37** | t1pks-nrps | GCF_98 | nrps |
| **GCF_38** | t1pks-cf_saccharide-nrps | GCF_99 | nrps |
| **GCF_39** | nrps | GCF_100 | nrps |
| **GCF_40** | nrps | GCF_101 | nrps |
| **GCF_41** | nrps | GCF_102 | nrps |
| **GCF_42** | nrps | GCF_103 | nrps |
| **GCF_43** | nrps | GCF_104 | nrps |
| **GCF_44** | nrps | GCF_105 | nrps |
| **GCF_45** | nrps | GCF_106 | nrps |
| **GCF_46** | nrps | GCF_107 | nrps |
| **GCF_47** | nrps | GCF_108 | nrps |
| **GCF_48** | nrps | GCF_109 | nrps |
| **GCF_49** | nrps | GCF_110 | nrps |
| **GCF_50** | nrps | GCF_111 | nrps |
| **GCF_51** | hserlactone-t1pks-nrps | GCF_112 | nrps |
| **GCF_52** | cf_saccharide-nrps | GCF_113 | nrps |
| **GCF_53** | transatpks | GCF_114 | nrps |
| **GCF_54** | transatpks | GCF_115 | nrps |
| **GCF_55** | transatpks | GCF_116 | nrps |
| **GCF_56** | transatpks | GCF_117 | hserlactone-transatpks-cf_fatty_acid |
| **GCF_57** | transatpks | GCF_118 | hserlactone-nrps |
| **GCF_58** | transatpks-t1pks-nrps | GCF_119 | cf_saccharide-nrps |
| **GCF_59** | transatpks-otherks | GCF_120 | cf_fatty_acid-t1pks |
| **GCF_60** | transatpks-cf_saccharide | GCF_121 | bacteriocin-lantipeptide |
| **GCF_61** | transatpks-cf_fatty_acid | GCF_122 | arylpolyene-nrps_(butunamide) |
